# Force-Regulated Spontaneous Conformational Changes of Integrins *α*_5_*β*_1_ and *α*_V_*β*_3_

**DOI:** 10.1021/acsnano.3c06253

**Published:** 2023-12-17

**Authors:** Yunfeng Chen, Zhenhai Li, Fang Kong, Lining Arnold Ju, Cheng Zhu

**Affiliations:** Woodruff School of Mechanical Engineering and Petit Institute for Bioengineering and Biosciences, Georgia Institute of Technology, Atlanta, Georgia 30332, United States; Department of Biochemistry and Molecular Biology and Department of Pathology, The University of Texas Medical Branch, Galveston, Texas 77555, United States; Shanghai Key Laboratory of Mechanics in Energy Engineering, Shanghai Institute of Applied Mathematics and Mechanics, School of Mechanics and Engineering Science, Shanghai University, Shanghai 200072, China; Woodruff School of Mechanical Engineering, Petit Institute for Bioengineering and Biosciences, and Coulter Department of Biomedical Engineering, Georgia Institute of Technology, Atlanta, Georgia 30332, United States; School of Biological Science, Nanyang Technological University, Singapore 637551, Singapore; Petit Institute for Bioengineering and Biosciences and Coulter Department of Biomedical Engineering, Georgia Institute of Technology, Atlanta, Georgia 30332, United States; School of Biomedical Engineering, The University of Sydney, Darlington, New South Wales 2008, Australia; Charles Perkins Centre, The University of Sydney, Camperdown, New South Wales 2006, Australia; Woodruff School of Mechanical Engineering, Petit Institute for Bioengineering and Biosciences, and Coulter Department of Biomedical Engineering, Georgia Institute of Technology, Atlanta, Georgia 30332, United States

**Keywords:** integrin, mechanobiology, molecular conformational change, biophysical modeling, molecular dynamics

## Abstract

Integrins are cell surface nanosized receptors crucial for cell motility and mechanosensing of the extracellular environment, which are often targeted for the development of biomaterials and nanomedicines. As a key feature of integrins, their activity, structure and behavior are highly mechanosensitive, which are regulated by mechanical forces down to pico-Newton scale. Using single-molecule biomechanical approaches, we compared the force-modulated ectodomain bending/unbending conformational changes of two integrin species, *α*_5_*β*_1_ and *α*_V_*β*_3_. It was found that the conformation of integrin *α*_5_*β*_1_ is determined by a threshold head-to-tail tension. By comparison, integrin *α*_V_*β*_3_ exhibits bistability even without force and can spontaneously transition between the bent and extended conformations with an apparent transition time under a wide range of forces. Molecular dynamics simulations observed almost concurrent disruption of ~2 hydrogen bonds during integrin *α*_5_*β*_1_ unbending, but consecutive disruption of ~7 hydrogen bonds during integrin *α*_V_*β*_3_ unbending. Accordingly, we constructed a canonical energy landscape for integrin *α*_5_*β*_1_ with a single energy well that traps the integrin in the bent state until sufficient force tilts the energy landscape to allow the conformational transition. In contrast, the energy landscape of integrin *α*_V_*β*_3_ conformational changes was constructed with hexa-stable intermediate states and intermediate energy barriers that segregate the conformational change process into multiple small steps. Our study elucidates the different biomechanical inner workings of integrins *α*_5_*β*_1_ and *α*_V_*β*_3_ at the submolecular level, helps understand their mechanosignaling processes and how their respective functions are facilitated by their distinctive mechanosensitivities, and provides useful design principles for the engineering of protein-based biomechanical nanomachines.

## Graphical Abstract

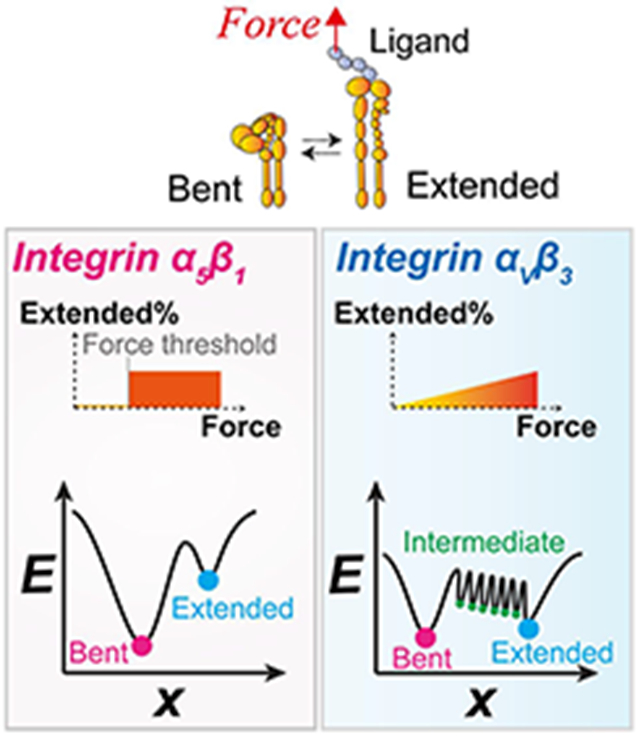

Integrins are a family of heterodimeric transmembrane molecules on the surface of nearly all cells. By mediating cell–cell/matrix adhesion and bidirectional transmembrane mechanosignal transduction, integrins play key roles in cellular functions, regulating cell attachment, migration, proliferation, differentiation, and more,^1^ while dysregulation of integrins is associated with diseases such as cancer, immune disorders and thrombosis.^2^ Integrins are often targeted for developing biomaterials for enhancing tissue and bone regeneration, wound healing, and device integration, and they have also inspired nanoparticles and nanomedicines for cancer diagnosis and treatment.^3-5^ In this context, it becomes crucial to understand the mechanosensitivity of integrins, because it not only mediates how cells interact with the (patho)physiological environment, but also critically affects cells’ compatibility and interaction with the biophysical properties of therapeutic tools and agents. For instance, the elasticity of nanoparticles has been shown to affect their *in vivo* localization and therapeutic efficacy.^6,7^ Of the 24 integrin species currently known, integrins *α*_5_*β*_1_ and *α*_V_*β*_3_ are used by a variety of cells to bind the extracellular matrix (ECM) and form focal adhesion. However, their functions are distinct: *α*_5_*β*_1_ molecules translocate laterally and cluster to support firm adhesion and cell spreading, whereas *α*_V_*β*_3_ molecules remain relatively stationary in focal adhesion and mediate early stage mechanotransduction and rigidity sensing.^8-10^ The molecular basis of such functional distinction is unclear, which was vaguely suggested to be related to the structural differences in *α*_5_*β*_1_ and *α*_V_*β*_3_ ectodomains.^9,10^

Force modulates the properties and functions of certain proteins by inducing conformational changes, such as coiling/uncoiling, zipping/unzipping, and folding/unfolding. We previously showed that cell surface *α*_L_*β*_2_ and *α*_V_*β*_3_ integrins undergo force-modulated conformational changes, such that force facilitates unbending but suppresses bending, shifting the conformational equilibrium toward extension.^11,12^ From a mechanical perspective, it is surprising that integrins can spontaneously bend against a wide range of forces. It is intuitive that a head-to-tail tension can facilitate a bent integrin to unbend regardless of which conformation is more stable prior to force application, because force can tilt the energy landscape and allow the extended conformation to become more stable, if this is not already the case in the absence of force. However, even if the bent conformation is more stable, its spontaneous bending against a tensile force is still counterintuitive when the tension is as high as 10–20 pN. The mechanical work done by the integrin to bend back under a linearly increasing force is comparable to the free energy of biotin–avidin binding (~35 $k_{B}T$),^13^ one of the strongest noncovalent interactions, and much greater than the environmental thermal agitation (0.5 $k_{B}T$).

Our previous experiments on force-modulated integrin bending and unbending were performed on living cells,^11,12^ where cell activity can regulate integrin conformational changes biologically. It seems natural to hypothesize that it is the cell that provides a “deactive energy” to bend the integrin against force. However, it is difficult to envision how this presumably cell-provided energy is converted into mechanical work to power the bending of the extended integrin, which occurs distally from the cell surface. To test this hypothesis, we used two force spectroscopic techniques to perform single-molecule experiments on purified integrins *α*_5_*β*_1_ and *α*_V_*β*_3_. While both integrins were able to undergo spontaneous bending and unbending under force, their conformational changes exhibited distinctive mechanical and kinetic properties, which may be related to their distinctive structures.^14^ Specifically, the conformation of integrin *α*_5_*β*_1_ was mostly bent in Ca^2+^ and mostly extended in Mn^2+^, suggesting a canonical energy landscape with a deep energy well that traps the integrin in the bent state in Ca^2+^ and the extended state in Mn^2+^. Force could tilt the energy landscape to shift the system in Ca^2+^ to bistability such that integrin *α*_5_*β*_1_ would abruptly transition back-and-forth between the bent and extended states. In contrast, integrin *α*_V_*β*_3_ might take either bent or extended conformation in both Ca^2+^/Mg^2+^ and Mn^2+^ conditions, and can undergo spontaneous bending and unbending with slow kinetics under a wide range of tensile forces similar to cell surface *α*_V_*β*_3_ integrins,^11^ falsifying our “biological energy” hypothesis and suggesting a physical mechanism. To explain the unusual behaviors of integrin *α*_V_*β*_3_, we developed a multistate conformational energy landscape for this integrin, which was supported by molecular dynamics simulations and could fit our experimental data well. The different mechanosensitivities of integrins *α*_5_*β*_1_ and *α*_V_*β*_3_ likely underlie their distinct biological functions in cell mechanosensing, which should help guide the development of more human compatible nanotherapeutics. The finding that integrin *α*_V_*β*_3_ can spontaneously bend and unbend under a wide range of mechanical forces without cell environment or the supply of external energy provides inspirational design concepts for protein-based biomechanical nanomachines.

## RESULTS/DISCUSSION

### Directly Observing Single Integrin *α*_5_*β*_1_ Unbending and Bending.

**Figure 1.**
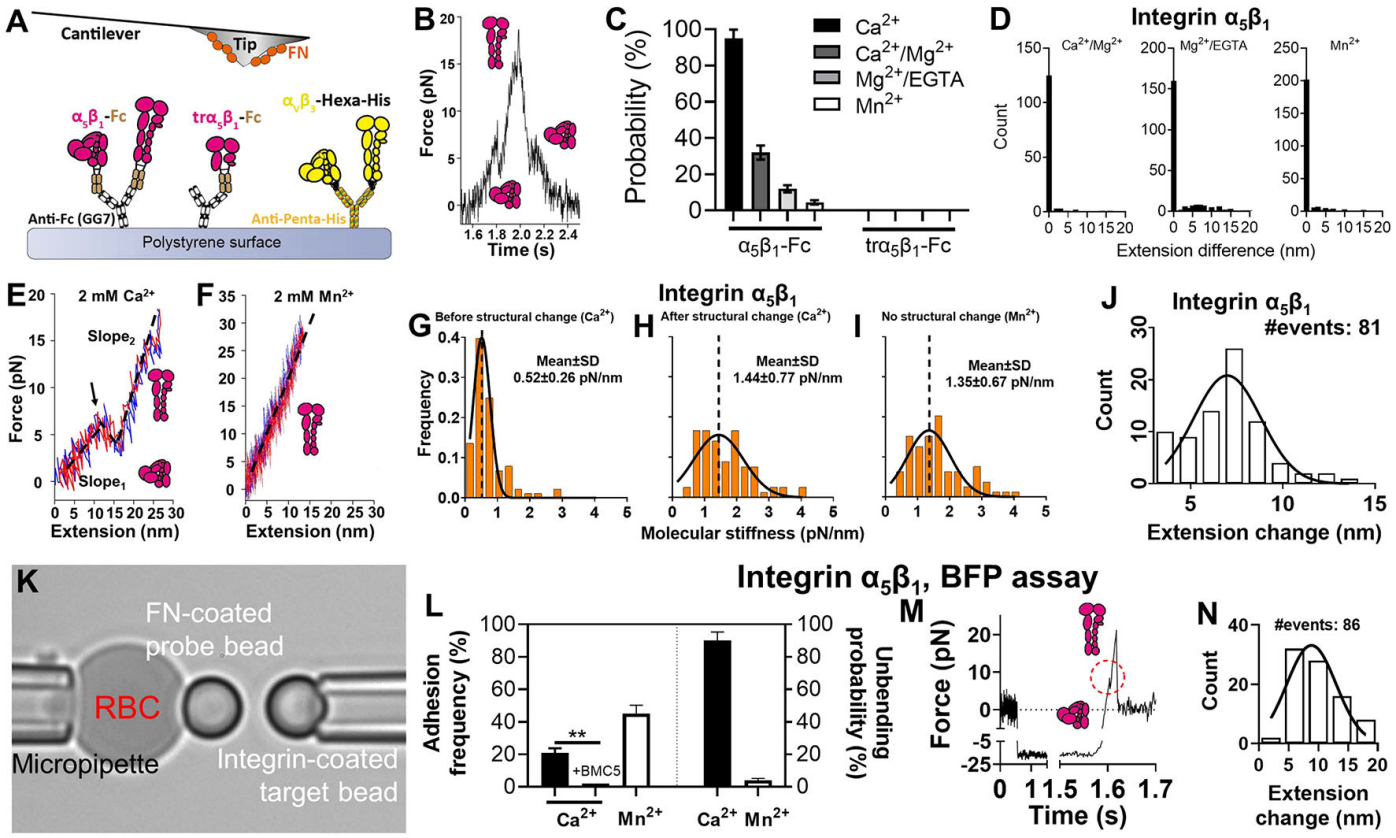
Observing and characterizing force-modulated integrin *α*_5_*β*_1_ unbending and bending. A. Superimposition of AFM experimental setups for integrins *α*_5_*β*_1_ and *α*_V_*β*_3_. Recombinant integrin *α*_5_*β*_1_, truncated integrin *α*_5_*β*_1_, and integrin *α*_V_*β*_3_ were respectively immobilized on a polystyrene surface using mAbs GG7 (anti-Fc) or anti-Hexa-Histidine. Here and in all following figures, integrins *α*_5_*β*_1_ and *α*_V_*β*_3_ are respectively colored by magenta and yellow. B. A representative AFM force vs time trace of a loading–unloading cycle on an integrin *α*_5_*β*_1_─FN bond. Two “kinks”, one in the loading and the other in the unloading phase, respectively represent integrin unbending and bending. C. Mean ± standard error of the probability of observing structural changes in integrin *α*_5_*β*_1_ or tr*α*_5_*β*_1_ in force loading–unloading processes in different metal ion conditions. D. Distribution of the difference of the integrin *α*_5_*β*_1_─FN complex molecular length before and after a full force loading–unloading cycle. E,F. Representative force vs extension curves of loading (*red*) and unloading (*blue*) in Ca^2+^ (E) and Mn^2+^ (F). The loading and unloading traces were linearly fitted (*black dashed lines*) to evaluate molecular stiffness, which shows in (E) two distictive stiffness values (*Slope*_1_, *Slope*_2_) exist for the bent and extended integrin *α*_5_*β*_1_, respectively. G-I. Histograms of the integrin *α*_5_*β*_1_─FN complex stiffness before (G) and after (H) unbending in Ca^2+^, and with no visible structural change in Mn^2+^ (I), and their respective Gaussian distribution fits (mean and standard deviation (SD) annotated). J. Histogram of AFM-measured integrin *α*_5_*β*_1_ head-to-tail molecular extension change due to unbending in Ca^2+^. K. BFP photomicrograph. L. Mean ± standard error of adhesion frequency (*left*) and *α*_5_*β*_1_ unbending probability (*right*) in Ca^2+^ and Mn^2+^ in BFP assay. The integrin *α*_5_*β*_1_-blocking monoclonal antibody (mAb), BMC5 eliminated most adhesion in Ca^2+^. M. Representative BFP force vs time trace of a force ramp cycle on an integrin *α*_5_*β*_1_─FN bond. An unbending event is highlighted in the red circle. N. Histogram of BFP-measured *α*_5_*β*_1_ head-to-tail molecular extension change due to unbending in Ca^2+^.

Using the atomic force microscopy (AFM), we tested whether mechanical force could induce conformational changes of integrin *α*_5_*β*_1_ independent of cell regulation. Recombinant integrin *α*_5_*β*_1_ ectodomain with a human IgG Fc tag at the tail (*α*_5_*β*_1_-Fc) was captured on a polystyrene surface (Figure 1A), and driven to touch the fibronectin module III domains 7–10 (abbreviated as FN, containing both the RGD sequence and synergistic site^15^) adsorbed on a cantilever tip to allow for bond formation. As has been confirmed in our previous work, the binding events measured by this experimental setup were predominantly mediated by specific interactions of *α*_5_*β*_1_-Fc (and tr*α*_5_*β*_1_-Fc used below) with FN^16^. A tensile force was loaded on each integrin *α*_5_*β*_1_─FN bond, which was ramped by retracting the polystyrene surface until reaching 20 pN, and then unloaded to 0 pN at the same rate (Figure 1B). Inspection of the force vs time traces often reveals a clearly visible kink in the middle of both the loading and unloading phases, where the slope of the curve suddenly drops from positive to zero or even negative in the loading phase and abruptly jumps from negative to zero or even positive in the unloading phase (Figure 1B). These kinks are clear indications of protein conformational changes such as unfolding–refolding.^17^

To identify the origin of these conformational changes, we first replaced *α*_5_*β*_1_-Fc with a truncated construct that contains only integrin *α*_5_*β*_1_ headpiece (tr*α*_5_*β*_1_-Fc; Figure 1A). In all four cation conditions—2 mM Ca^2+^ (Ca^2+^), 1 mM Ca^2+^ plus 1 mM Mg^2+^ (Ca^2+^/Mg^2+^), 1 mM Mg^2+^ plus 1 mM EGTA (Mg^2+^/EGTA) and 2 mM Mn^2+^ (Mn^2+^)—that favor different integrin conformations, the conformational changes seen in the full-length *α*_5_*β*_1_-Fc were no longer observed (Figure 1C), indicating that these conformational changes are from the *α*_5_*β*_1_ ectodomain but not the recombinant Fc tail, GG7 or FN, and require the integrin *α*_5_*β*_1_ tailpiece. Second, such conformational changes occurred progressively less frequently as the cation composition changed to those that activate integrins more and more potently, resulting in a frequency hierarchy of Ca^2+^ > Ca^2+^/Mg^2+^ > Mg^2+^/EGTA > Mn^2+^ (Figure 1C). This suggests the observed structural lengthening/shortening events to be those of integrin unbending/bending, which explains the frequency hierarchy: the activating cation conditions facilitate more integrins to adopt the extended conformation, leaving less integrins in the bent conformation capable of unbending. Third, in all cation conditions, the structural lengthening in the loading phase was almost always ensued by a structural shortening in the unloading phase. By plotting force against the molecular extension of the bond, we found that the molecular complex fully recovers to its original length at the end of the loading–unloading cycle with no hysteresis (Figure 1D) and the loading and unloading phases largely overlap (Figure 1E), suggesting that the conformational changes are highly reversible and ruling out the alternative interpretation that they represent irreversible structure denaturation. Fourthly, the slope of the force–extension curve, which represents molecular stiffness, was seen to always increase after a structural lengthening (Figure 1E,G,H) and decrease after a structural shortening. The curve segments before and after the structural extension were both well-fitted by a linear model, while the worm-like chain (WLC) model did not render better results (Supp. Figure 1A). Furthermore, the slope of the force–extension curve remained constant in the absence of a structural change (Supp. Figure 1B), ruling out the possibility that the molecular stiffening was caused by the WLC nonlinear response. Together, these results indicate that *α*_5_*β*_1_ becomes stiffer after the structural lengthening and softer after the structural shortening. This is consistent with our previous observations on multiple other integrin species that integrins are stiffer in their extended conformation than in the bent conformation.^11,12,18^ Using molecular stiffness as a signature readout of integrin conformation, we found that the value of the post-extension stiffness is comparable to integrins in Mn^2+^ showing no structural changes (Figure 1F,H,I), which agrees with our hypothesis that integrins would be nearly unable to unbend or bend in this cation condition, because Mn^2+^ has already activated most of the integrins to the extended conformation. Finally, the molecular extension change due to structural lengthening centers around 7.5 nm (Figure 1J), which is comparable to the head-to-tail length increase of an unbending integrin *α*_5_*β*_1_ characterized by theoretical modeling.^19^ The broad distribution of the extension change was most likely due to the intermolecular variation in the headpiece/tailpiece angle of both the bent and extended conformations in different *α*_5_*β*_1_ molecules.^20,21^

To further validate our discovery, a recombinant integrin *α*_5_*β*_1_ ectodomain fused with a polyhistidine tag at the C-terminus (*α*_5_*β*_1_-Poly-His) was tested on another force spectroscopy technique, Biomembrane Force Probe (BFP). The BFP setup consisted of a micropipette-aspirated human red blood cell (RBC) with a streptavidin (SA) and FN cofunctionalized probe bead attached to its apex to serve as an ultrasensitive force transducer (Figure 1K, *left*). A bead coated with *α*_5_*β*_1_-Poly-His was aspirated by an opposing micropipette (Figure 1K, *right*) and driven to repeatedly contact the probe bead to induce integrin *α*_5_*β*_1_─FN bond formation. The specificity of the bonds was confirmed with the fact that addition of the mAb BMC5 eliminated most of the binding events (Figure 1L). Similar to our observation in the AFM assay, a “kink” was observed in the force vs time traces when the bonds were ramped by force (Figure 1M), which was observed much more frequently in Ca^2+^ condition than in Mn^2+^ (Figure 1L). The molecular extension change induced by unbending centers at 8.8 nm (Figure 1N), which was slightly longer than the value collected by AFM. This was likely due to the softer force transducer of BFP that does not favor the observation of small molecular extensions in the ramping phase, which could cause a bias in the event detection. Together, these results indicate that real-time integrin *α*_5_*β*_1_ unbending and bending events could be observed using our force spectroscopy approaches.

### Directly Observing Single Integrin *α*_V_*β*_3_ Bending and Unbending.

The biophysical characteristics of force-modulated integrin *α*_5_*β*_1_ unbending and rebending are different from those previously characterized for integrins *α*_L_*β*_2_ and *α*_V_*β*_3_ on the cell surface. The force range within which structural change events could be observed is quite narrow for integrin *α*_5_*β*_1_ (<10 pN, Figure 1B,E; also see Figure 5A-D below) but much wider for *α*_L_*β*_2_ and *α*_V_*β*_3_ (up to 40 pN).^11,12^ On the other hand, kinetics rates of the conformational changes can be quantified by two parameters: time-to-switch ($t_{0\pm }$) is the waiting time required for the conformational switch to occur, while switching time ($t_{\text{sw}\pm }$) is the time taken for the conformational switch from the start to finish.^11,12^ Using these definitions, we found that the kinetics are much more rapid for integrin *α*_5_*β*_1_ than cell surface integrins *α*_L_*β*_2_ and *α*_V_*β*_3_ (e.g., $t_{0-}$ at 5 pN is ~0.02 s vs 2–3 s).^11,12^ A hypothetical explanation for the different (un)bending behaviors observed here and previously might be the absence of cell regulation for integrin *α*_5_*β*_1_. To test this hypothesis, we studied the conformational changes of integrin *α*_V_*β*_3_ ectodomain bound to FN, so as to allow the direct comparison of integrins *α*_5_*β*_1_ and *α*_V_*β*_3_ as purified proteins. The AFM approach was first applied. Despite that control experiments confirmed the detection of integrin *α*_V_*β*_3_─FN specific binding, no “kink”, i.e., sudden slope change in the force-time curve, was observed over hundreds of force loading–unloading events; clamping the integrin *α*_V_*β*_3_─FN bonds under a constant force or applying cyclic forces did not yield any kink type of conformational changes either.

**Figure 2.**
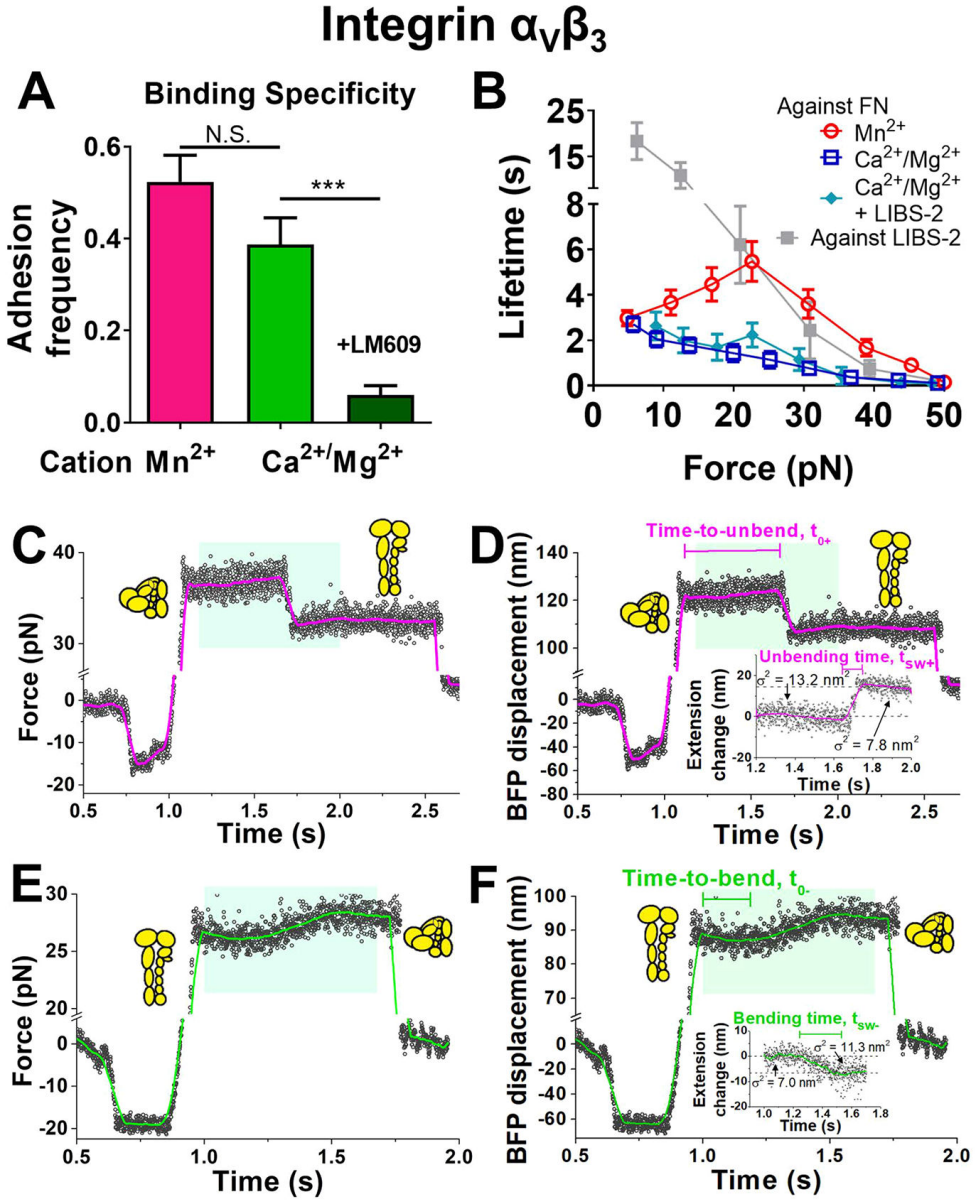
The observation of force-regulated integrin *α*_V_*β*_3_ unbending and bending by BFP. A. The adhesion frequency of integrin *α*_V_*β*_3_─FN binding in Mn^2+^ and Ca^2+^/Mg^2+^ conditions. The addition of mAb LM609 blocked most of the adhesion events in Ca^2+^/Mg^2+^. B. Mean ± s.e.m. of lifetime vs force of single integrin *α*_V_*β*_3_─FN bonds in indicated conditions or integrin *α*_V_*β*_3_– LIBS-2 bonds. C–F. Representative BFP force vs time (C,E) and displacement vs time (D,F) traces respectively showing an integrin unbending (C,D) and bending (E,F) event in the position-clamp phase, along with cartoons depicting different integrin *α*_V_*β*_3_ conformations before and after (un)bending. Panels D and F are respectively converted from Panels C and E, where BFP displacement is calculated as Force/*k*_RBC_ (RBC spring constant). The data (*points*) is smoothened using the Savitzky-Golay method (*curves*) to obtain a higher force resolution. Inserts in panels D and F: detailed views of the conformational changes within the cyan-shaded windows that convert the BFP displacement to the integrin *α*_V_*β*_3_ extension change, with standard deviations of the signals, *σ*, indicated as a measure of thermal fluctuation before and after the (un)bending. Definitions of time-to-switch and switching time are indicated.

Cell surface integrin *α*_V_*β*_3_ (un)bending events were previously observed using BFP^11^. We reasoned that the observation of integrin *α*_V_*β*_3_ conformational changes might not be favored by the stiff AFM cantilever but favored by the soft BFP force sensor (spring constants ~3 vs ~0.3 pN/nm), because a 10-nm head-to-tail length change caused by integrin *α*_V_*β*_3_ bending would result in a ~30 pN force increase in the AFM, which would severely inhibit bending, but only ~3 pN force increase in the BFP, which would not. We thus used the BFP for testing, wherein the probe bead was again cofunctionalized with SA and FN, while the target bead was coated with recombinant *α*_V_*β*_3_ protein. Binding specificity was confirmed by an integrin *α*_V_*β*_3_-blocking mAb, LM609, which abolished most of the binding events (Figure 2A).

After the FN coating was titrated on probe beads to lower the adhesion frequency to 20%, a necessary condition for most binding events to be mediated by single bonds,^22^ integrin *α*_V_*β*_3_ was then interrogated under both Ca^2+^/Mg^2+^ and Mn^2+^ conditions using distance-clamping assay:^11^ the integrin *α*_V_*β*_3_─FN bond was first pulled to a certain force level, and the target bead was then clamped at the position until bond dissociation. The bonds could sustain a wide range of forces, with lifetimes much longer in Mn^2+^ than in Ca^2+^/Mg^2+^, consistent with the activating role of Mn^2+^ (Figure 2B). Unlike the purified integrin *α*_5_*β*_1_─FN interaction that forms catch-slip bonds not only in Mn^2+^, but also in Ca^2+^/Mg^2+^ and Mg^2+^/EGTA,^16^ the purified integrin *α*_V_*β*_3_−FN interaction formed a catch-slip bond in Mn^2+^ but a slip-only bond in Ca^2+^/Mg^2+^. This slip-only bond indicates the limited effect of a sustained force to strengthen integrin *α*_V_*β*_3_ bonding to FN, which agrees with our previously reported weak integrin *α*_V_*β*_3_─FN catch-slip bond on the cell surface.^11^

**Figure 3.**
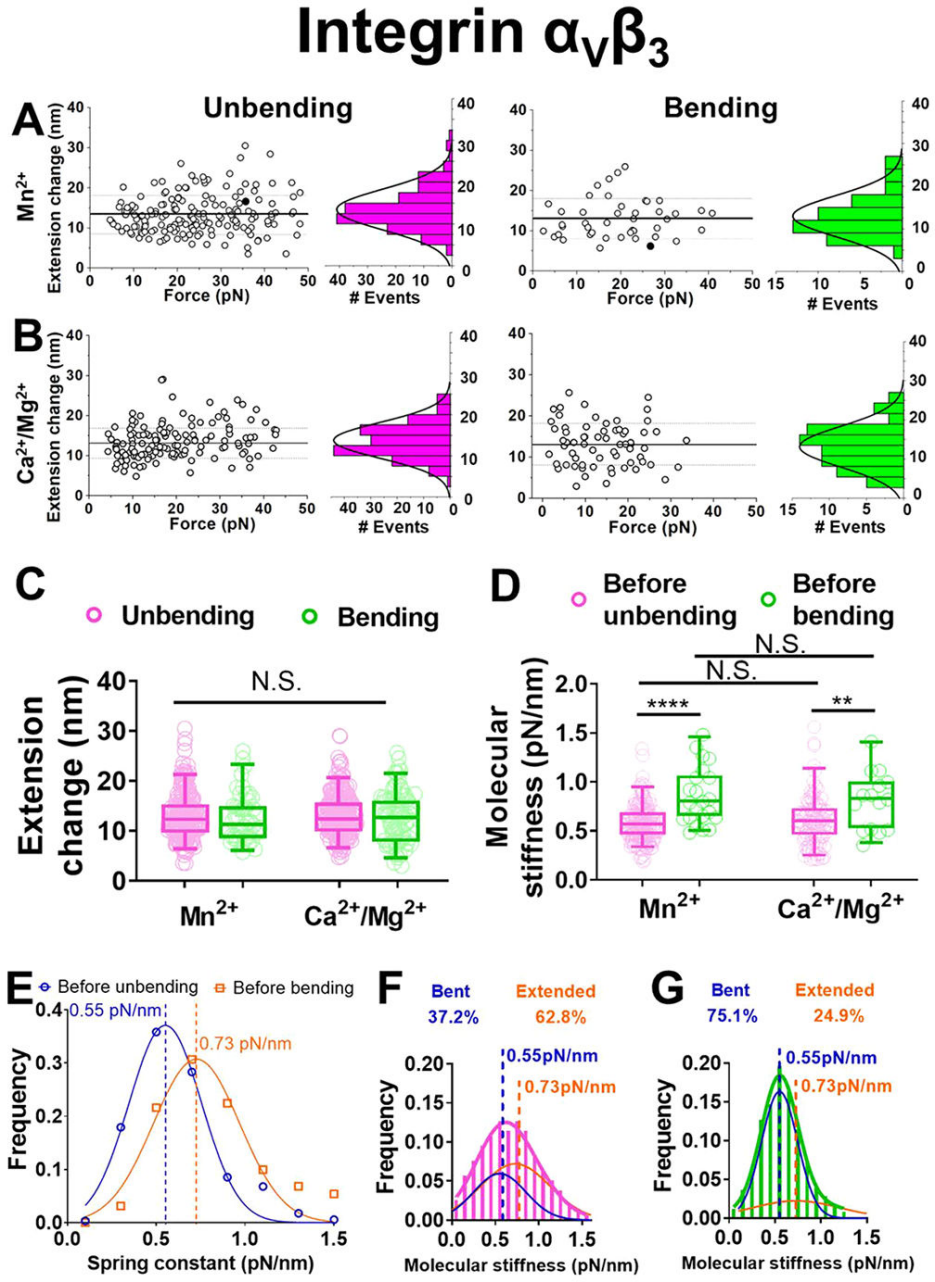
Characterization of force-regulated integrin *α*_V_*β*_3_ unbending and bending by BFP. A,B. Scatter plots, histograms (*bars*) and Gaussian fits (*curves*) of integrin *α*_V_*β*_3_ extension changes due to unbending (*left*) and bending (*right*) in Mn^2+^ (A) and Ca^2+^/Mg^2+^ (B). The two solid dots in (A) respectively correspond to the representative unbending and bending events depicted in Figures 2D,E and 2F,G. C. Data (*points*) and the median and 5–95 percentiles (box and whisker) of integrin *α*_V_*β*_3_ extension changes due to unbending and bending in Mn^2+^ and Ca^2+^/Mg^2+^. D. Data (*points*) and the median and 5–95 percentiles (box and whisker) of the integrin *α*_V_*β*_3_─FN molecular stiffness before unbending events and before bending events. N.S. = not significant; ** *p* < 0.01; **** *p* < 0.0001, assessed by one-way ANOVA. E. Fitting the integrin *α*_V_*β*_3_─FN molecular stiffness before unbending and before bending with Gaussian distribution to respectively acquire the average molecular stiffness associated with bent and extended integrins. F,G. Fitting the integrin *α*_V_*β*_3_/FN molecular stiffness in Mn^2+^ (F) and Ca^2+^/Mg^2+^ (G) with dual-Gaussian distribution to calculate the proportions of BFP-detected integrins in bent and extended conformations. The means of the two Gaussian distributions, respectively associated with bent and extended integrin *α*_V_*β*_3_, were derived from (E).

In the clamping phase of some lifetime measurements, we observed integrin *α*_V_*β*_3_ unbending or bending events, respectively signified by a concurrent decrease in the mean force and force fluctuation or a concurrent increase in the mean force and force fluctuation (Figure 2C-F; Supp. Table 1), which are clearly distinguishable from formation of an additional bond (signified by an increase in the force and a decrease in thermal fluctuation) and dissociation of a bond from a multibond adhesion (signified by a decrease in the force and an increase in thermal fluctuation).^11,12^ In most cases, instead of successive back-and-forth transitions, only a single conformational change event could be observed in the distance-clamp cycle, which is likely due to the limitation of integrin *α*_V_*β*_3_─FN bond lifetimes that were too short to provide a long enough observation window to overcome the slow kinetics of integrin *α*_V_*β*_3_ conformational changes (shown below). Unlike purified integrin *α*_5_*β*_1_ and consistent with cell surface integrin *α*_V_*β*_3_, the conformational changes of purified integrin *α*_V_*β*_3_ occurred under a wide range of forces (Figure 3A,B) with relatively long time-to-switch ($t_{0+}$ and $t_{0-}$ respectively for unbending and bending) and switching time ($t_{\text{sw}+}$ and $t_{\text{sw}-}$ respectively for unbending and bending)^11^ (cf. Figure 2C-F). Such unusually slow kinetics ruled out the alternative possibility that these conformational changes were protein domain unfolding/refolding events, which are generally abrupt (e.g., talin^23^) due to the involved local secondary structure being relatively simple. Replacing FN on the probe beads with LIBS-2, a mAb that binds the *α*_V_*β*_3_ *β*TD domain at its tailpiece,^24^ abolished the above signature signals for integrin conformational changes (Supp. Table 1) despite the long lifetimes (Figure 2B), further ruling out the alternative possibility that the putative bending/unbending events are due to multiple bond rupture/formation or instrumental drift. Adding high-concentration LIBS-2 to the solution, which stabilizes *β*_3_ integrins in the extended conformation,^25^ also eliminated all bending events (Supp. Table 1). Interestingly, LIBS-2 treatment did not significantly alter the integrin *α*_V_*β*_3_─FN bond type and lifetimes in Ca^2+^/Mg^2+^ (Figure 2B), indicating that integrin extension and catch-slip bond formation are decoupled. During (un)bending, the change in the RBC elongation (Figure 2D,F, *cyan shaded areas*) is equal to the change in the integrin head-to-tail length.^11,12^ These length changes of both unbending and bending events follow a single-Gaussian distribution (Figure 3A,B) with an indistinguishable average value of ~13 nm in both Ca^2+^/Mg^2+^ and Mn^2+^ conditions (Figure 3C), agreeing with our previous observations of cell surface integrin *α*_V_*β*_3_ bending/unbending events and with our MD simulation results on integrin *α*_V_*β*_3_ unbending.^11,26^ The length changes observed here on integrin *α*_V_*β*_3_ are much longer than *α*_5_*β*_1_ (Figure 1J,N), which is primarily due to the difference between the two integrin species. Bent integrin *α*_V_*β*_3_ adopts a highly compact structure with a headpiece-tailpiece angle of ~40° (refs 27, 28; also shown in Figure 6E below). In contrast, the bent conformation of integrin *α*_5_*β*_1_ is less tight where the headpiece-tailpiece angle reaches 71°–93° (ref 21; also shown in Figure 6A below), therefore shortening the traveling distance of its headpiece during conformational changes.

Furthermore, the stiffness of the integrin *α*_V_*β*_3_─FN complex is lower before unbending than before bending (Figure 3D), consistent with the signature integrin stiffening upon unbending.^11,12,18^ Since the stiffness depends only on the conformation but not the cation condition (Figure 3D), we pooled data from both cation conditions together to examine the stiffness distributions for the bent and extended integrins, finding their respective means and standard deviations of 0.55 ± 0.20 and 0.73 ± 0.24 pN/nm (Figure 3E), comparable to the values previously measured from cell surface *α*_V_*β*_3_ (ref 11). Moreover, we plotted the histograms of additional stiffness measurements from each cation condition, regardless of whether integrin (un)bending events were observable, and fitted each by a dual-Gaussian distribution using 0.55 and 0.73 pN/nm as the two means to calculate the proportions of integrins in the bent and extended states. We found that, of those *α*_V_*β*_3_ integrins that formed bonds, 62.8% were in the extended conformation in Mn^2+^, but 24.9% were in the extended conformation in Ca^2+^/Mg^2+^ (Figure 3F,G), consistent with the activating role of Mn^2+^. More importantly, these results confirm the previous observation that integrin *α*_V_*β*_3_, unlike *α*_5_*β*_1_, is already bistable under zero force.^28^ Overall, the data confirm that purified integrin *α*_V_*β*_3_ protein can spontaneously transition between the bent and extended conformations under a wide range of forces in the absence of cellular regulation or biological energy supply.

Integrin-mediated mechanosignaling was conventionally believed to require either integrins to cluster, so as to trigger rearrangement of cytoskeletal structure,^29,30^ or alternatively, prior inside-out signaling to unbend the integrin for activation and ligand binding (“switch-blade” model^31^), and/or to activate intracellular scaffold proteins (e.g., talin in “molecular clutch” model^32^) for signal transduction. Our findings on integrins *α*_5_*β*_1_ and *α*_V_*β*_3_, together with previous echoing works,^11,12^ indicated that bent integrins can also bind to ligands and that integrin unbending can be solely modulated by mechanical force. These shreds of evidence suggest an additional mechanism that allows a single inactive integrin to initiate outside-in mechanosignaling without prior inside-out signaling, wherein the unbending conformational change propagates intracellularly to induce integrin tailpiece separation,^33,34^ integrin cluster rearrangement^14,35^ and/or the association of cytoplasmic proteins.^14^

### Integrin *α*_V_*β*_3_ Showed No Cyclic Mechanical Reinforcement Effect.

**Figure 4.**
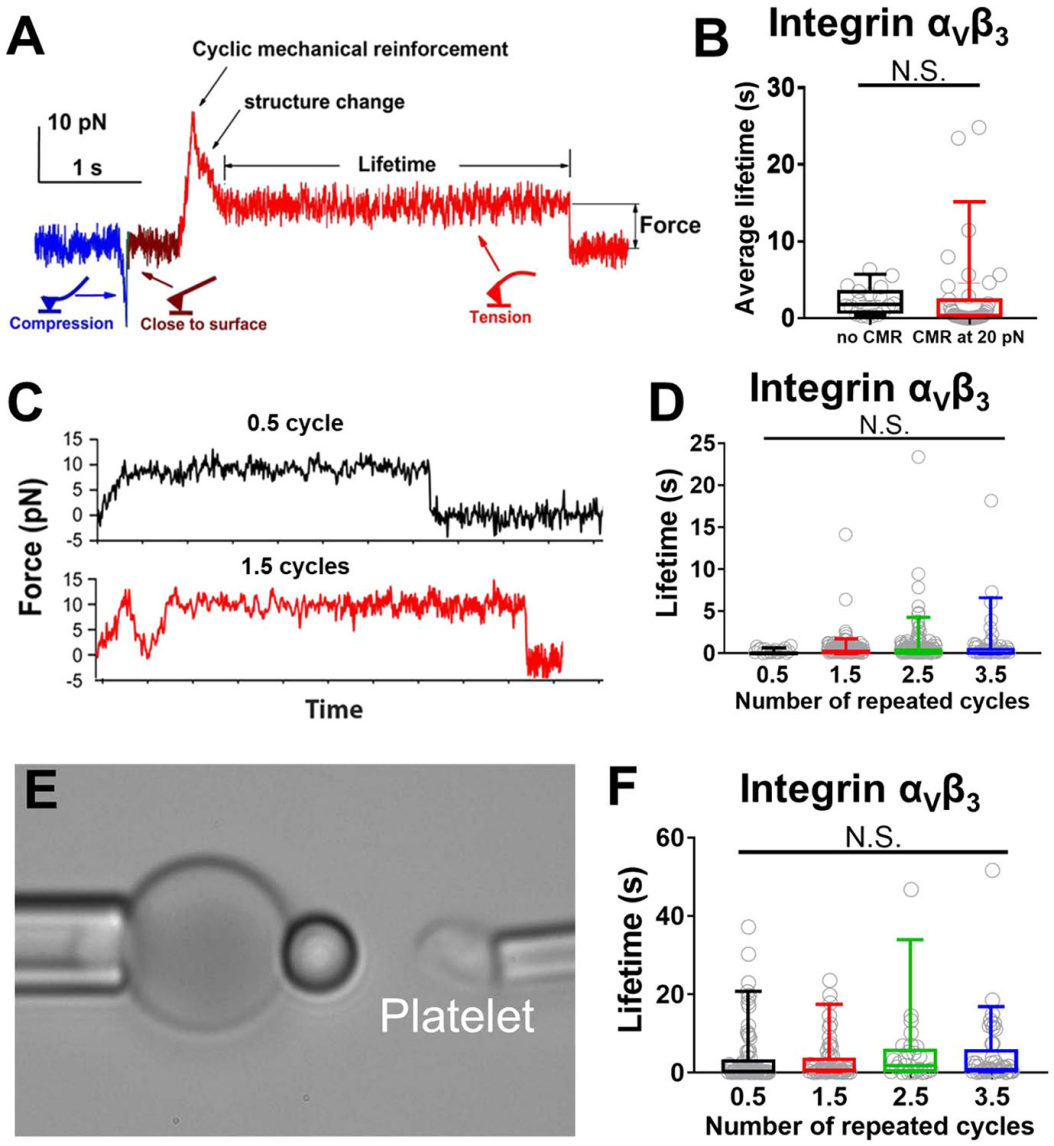
Measuring cyclic mechanical reinforcement (CMR) of integrin *α*_V_*β*_3_ using AFM and BFP. In both systems the ligand coating was titrated to reach infrequent adhesion (~20%), a necessary condition for most adhesion events to be mediated by single bonds. A. A representative AFM force vs time trace showing a CMR with one loading–unloading cycle with a ~20 pN peak force followed by bond lifetime measurement at ~5 pN, which was used to generate the data in the right group of panel (B). The cartoons indicated how the cantilever would be bent in different segments of the data curve. B. Data (*points*) and the median and 5- 95 percentiles (box and whisker) of integrin *α*_V_*β*_3_─FN bond lifetimes measured after a singlecycle CMR (*red*, exemplified in panel (A)) or without CMR (*black*, exemplified in panel (C), *top*). C. Two representative AFM force vs time traces showing integrin *α*_V_*β*_3_ lifetime measurements of a bond with 0.5 (*top*) and 1.5 (*bottom*) loading–unloading cycle before clamping at the peak force, which were used to generate the data in the first two groups in (D). D. Data (points) and the median and 5–95 percentiles (box and whisker) of integrin *α*_V_*β*_3_─FN bond lifetimes measured after the indicated numbers of CMR cycles. E. BFP photomicrograph showing the experiment setup used to generate the data in (F), where a platelet aspirated by an opposing micropipette acted as the target. F. Data (*points*) and the median and 5–95 percentiles (box and whisker) of platelet integrin *α*_V_*β*_3_─FN bond lifetimes measured after the indicated numbers of CMR cycles using the BFP shown in (E).

Integrin spontaneous unbending and bending respectively decrease and increase its ligand binding force (Figure 2C,E), which may help strengthen the bonds through a mechanism called “cyclic mechanical reinforcement” (CMR), where a cyclic force applied to a receptor–ligand bond greatly prolongs its lifetime. CMR was initially observed with integrin *α*_5_*β*_1_─FN bonds,^36^ but later also observed with actin–actin bonds.^37^ To test the CMR effect on integrin *α*_V_*β*_3_─FN bonds, we first used AFM as did previously on integrin *α*_5_*β*_1_─FN bonds.^36^ Once a bond was detected, two types of cyclic forces were applied: 1) one loading–unloading cycle that first peaks at 20 pN and then drops to and is held at 5 pN (Figure 4A); and 2) cyclic forces with zero, one, two or three complete loading–unloading cycles followed by ramping to and being clamped at a peak force of 10 pN (Figure 4C). Unexpectedly, neither type of cyclic forces prolonged *α*_V_*β*_3_─FN lifetimes, showing a lack of CMR effect (Figure 4B,D).

We also repeated the above experiments using BFP with integrin *α*_V_*β*_3_-expressing platelets as the target (Figure 4E). Inhibitory mAbs 10E5 and P1D6 were added to respectively block *α*_IIb_*β*_3_ and *α*_5_*β*_1_, two other FN-binding integrins on platelets, to ensure sole interaction of integrin *α*_V_*β*_3_ with FN^18^. The second type of force loading–unloading cycles was applied to integrin *α*_V_*β*_3_─FN bonds followed by ramping to and clamping at 10 pN. Despite the presence of cell environment, the bond lifetime of integrin *α*_V_*β*_3_ with FN was still not prolonged by cyclic forces (Figure 4F).

### Distinctive force-dependent kinetics of integrins *α*_5_*β*_1_ and *α*_V_*β*_3_ conformational changes.

**Figure 5.**
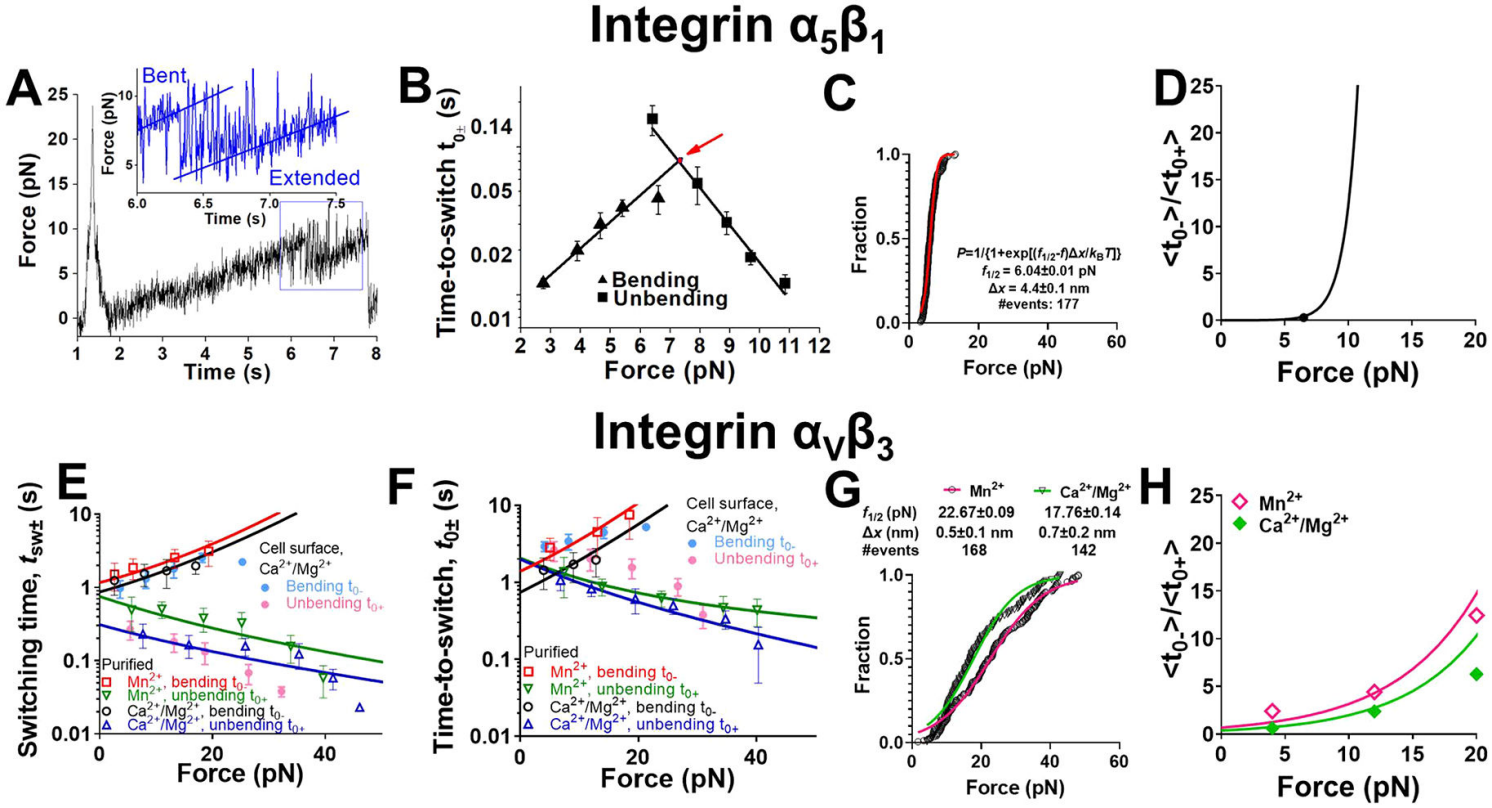
Force-modulated integrin *α*_5_*β*_1_ and *α*_V_*β*_3_ bending and unbending kinetics. A. A representative force vs time trace of applying slow ramping force on an integrin *α*_5_*β*_1_─FN bond after a single CMR cycle, which was measured by AFM in Ca^2+^ to exemplify reversible and consecutive unbending–bending events of integrin *α*_5_*β*_1_. Insert: zoom-in of the curve segment showing repeated bending-unbending events in a narrow force range near ~7 pN. B. Semilog plots of mean ± s.e.m., integrin *α*_5_*β*_1_ time-to-unbending $t_{0+}$ (*square*) and time-to-bending $t_{0-}$ (*triangle*) vs force data and their fits by the Bell model (*curves*). The two fitting curves intersect at 7.4 ± 0.6 pN and 0.076 ± 0.017 s (*arrow*). C. Cumulative histogram of integrin *α*_5_*β*_1_ unbending force distribution. The distribution was fitted by a theoretical model to derive the parameters of the energy landscape. The equation of the model and the derived parameters were denoted. D. Plot of $\langle t_{0-}\rangle$ to $\langle t_{0+}\rangle$ ratio of integrin *α*_5_*β*_1_ conformational changes vs force, calculated based on experimental data (*point*) and the model fitting in panel (C) (*curve*). E,F. Semilog plots of mean ± s.e.m., integrin *α*_V_*β*_3_ unbending time $t_{\text{sw}+}$ (E) or time-to-unbending $t_{0}+$ (F) (*hollow triangle* and *hollow inverted triangle*) and bending time $t_{\text{sw}-}$ (E) or time-to-bending $t_{0-}$ (F) (*hollow square and hollow circle*) vs force data measured in the indicated cation conditions, and their theoretical fits by the multistate model described in the text. The *R*^2^ values of the fittings are 0.95 and 0.96 for Mn^2+^ and Ca^2+^/Mg^2+^ conditions, respectively. Solid dots: mean ± s.e.m. $t_{0\pm }$ and $t_{\text{sw}\pm }$ vs force of cell surface integrin *α*_V_*β*_3_ unbending (*light magenta*) and bending (*light cyan*) events in Ca^2+^/Mg^2+^. G. Cumulative histogram of integrin *α*_V_*β*_3_ unbending force distribution with theoretical model fitting. H. Plots of $\langle t_{0-}\rangle$ to $\langle t_{0+}\rangle$ ratio of integrin *α*_V_*β*_3_ vs force measured under indicated cation conditions and their model fits.

The distinctive biophysical behaviors in the conformational changes of *α*_5_*β*_1_ and *α*_V_*β*_3_ integrins prompted us to analyze and compare the kinetics of their bending and unbending conformational changes as characterized by switching time ($t_{\text{SW}\pm }$) and time-to-switch ($t_{0\pm }$). We employed AFM to pull integrin *α*_5_*β*_1_ slowly (~1 nm/s) after performing CMR to strengthen its bond with FN, which prolonged the time for observation of repetitive unbending-bending cycles in a single binding event^36^ (Figure 5A), allowing us to collect ensembles of measurements for kinetic analysis. The bending and unbending processes were too fast to measure $t_{\text{SW}\pm }$ values (always beyond the temporal resolution of 1 ms of our AFM instrument) and the individual $t_{0\pm }$ values were highly fluctuating (Figure 5A, *inset*). Nevertheless, the average $\langle t_{0+}\rangle$ decreased exponentially, and $\langle t_{0-}\rangle$ increased exponentially, with increasing force f (Figure 5B), behaving as a typical slip bond and catch bond, respectively.^38^ We model the force-dependent $\langle t_{0+}\rangle$ and $\langle t_{0-}\rangle$ using the Bell equation^39^ (eq 1a) and its “catch bond counterpart” (eq 1b):

(1a) $$\langle t_{0+}\rangle =\langle t_{0+}l_{f=0}\rangle \text{exp}[-f{\Delta x}_{+}∕k_{B}T]$$

(1b) $$\langle t_{0-}\rangle =\langle t_{0-}l_{f=0}\rangle \text{exp}[f{\Delta x}_{-}∕k_{B}T]$$

where $k_{B}$ is the Boltzmann constant, T is absolute temperature, $\langle t_{0\pm }l_{f=0}\rangle$ are the respective values of $\langle t_{0\pm }\rangle$ at zero force, and ${\Delta x}_{\pm }$ respectively represent the distances from the top of the energy barrier to the bottoms of the energy wells of the bent (${\Delta x}_{+}$) and extended (${\Delta x}_{-}$) conformations in the energy landscape at zero force. Directly fitting eqs 1a and 1b to the respective $\langle t_{0+}\rangle$ and $\langle t_{0-}\rangle$ data in Figure 5B yielded excellent agreement and returned $\langle t_{0+}l_{f=0}\rangle =5.5\pm 2.9\;s$, ${\Delta x}_{+}=1.6\pm 0.16\;\text{nm}$, $\langle t_{0-}l_{f=0}\rangle =0.004\pm 0.0007$, and ${\Delta x}_{-}=2.4\pm 0.25\;\text{nm}$.

Since the reciprocal average time-to-bending and reciprocal average time-to-unbending are the kinetic rates of bending and unbending, respectively, we can calculate the bending equilibrium coefficient as a function of force by taking the ratio of eq 1b to eq 1a, which yields

(2) $$\frac{\langle t_{0-}\rangle }{\langle t_{0+}\rangle }=\frac{\langle t_{0-}l_{f=0}\rangle }{\langle t_{0+}l_{f=0}\rangle }\text{exp}(f\Delta x∕k_{B}T)\;\;=\text{exp}\left[\frac{f\Delta x-\text{ln}(\langle t_{0+}l_{f=0}\rangle ∕\langle t_{0-}l_{f=0}\rangle )}{k_{B}T}\right]$$

where $\Delta x={\Delta x}_{+}+{\Delta x}_{-}$. Let $f_{1∕2}$ be the force at which $\langle t_{0-}\rangle ∕\langle t_{0+}\rangle \;=\;1$, i.e., the force at which the time-to-unbending $\langle t_{0+}l_{f=f_{1∕2}}\rangle$ equals to the time-to-bending $\langle t_{0-}l_{f=f_{1∕2}}\rangle$. For $\alpha_{5}\beta_{1}$, Δx=4.0±0.3nm, and $f_{1∕2}=\text{ln}\;(\langle t_{0+}l_{f=0}\rangle ∕\langle t_{0-}l_{f=0}\rangle )∕\Delta x=7.4\pm 0.6\;\text{pN}$.

The definition of ${\left[\langle t_{0-}\rangle ∕\langle t_{0+}\rangle \right]}_{f=f_{1∕2}}=1$ predicts that near $f_{1∕2}$, integrin $\alpha_{5}\beta_{1}$ has an equal chance of residing in the bent and extended states. The value of $\langle t_{0+}l_{f=f_{1∕2}}\rangle =\langle t_{0-}l_{f=f_{1∕2}}\rangle =0.076$ s predicts that the integrin transitions rapidly back-and-forth between these two states. Such consecutive back-and-forth events with brief intermittent durations were indeed observed, but occurred at comparable frequencies only in a narrow force range (6–9 pN, Figure 5A). The probability of time during which the integrin stays in the extended state can be derived from eq 2:

(3) $$P=\frac{\langle t_{0-}\rangle }{\langle t_{0+}\rangle +\langle t_{0-}\rangle }={\left\{1+\text{exp}[(f_{1∕2}-f)\Delta x∕k_{B}T]\right\}}^{-1}$$

Here $f_{1∕2}$ is defined by the same formula but interpreted as the force at which the integrin has a 50–50 chance of staying in either the bent or extended state. We plotted the measured fraction of extension times (points) and the fitting of eq 3 (curve) to the data (Figure 5C), which showed excellent agreement and returned a slightly larger Δx=4.40±0.06nm and a slightly smaller $f_{1∕2}=6.04\pm 0.01\;\text{pN}$. The consistency between the values obtained by fitting eqs 1a and 1b to the data in Figure 5B and those by fitting eq 3 to the data in Figure 5C supports the quality of our data, the appropriateness of our model, and the robustness of the model parameters.

Across the $f_{1∕2}$ threshold, force quickly transitioned the integrin from the bent to extended conformation: as force increased from 4.3 to 10.5 pN, the dominant (>95%) population of integrin molecules rapidly changed from the bent to the extended conformation, which increased the population ratio of extended over bent integrins $\langle t_{0-}\rangle ∕\langle t_{0+}\rangle$ by 400-fold, corresponding to an average force sensitivity of >60-fold/pN (Figure 5D). Such a high force-sensitivity is due to the relatively large Δx value and agrees with a previous theoretical study inferring that integrin *α*_5_*β*_1_ unbending is ultrasensitive to force,^19^ reflecting nearly “digital” modulation of force on *α*_5_*β*_1_ conformation.

Compared with the conformational change kinetics of integrin *α*_5_*β*_1_, which were rapid and strongly force-dependent, the kinetics of integrin *α*_V_*β*_3_ conformational changes were slow and weakly force-dependent. Such characteristics were revealed by using the same approaches as above to analyze the counterpart data for integrin *α*_V_*β*_3_, which occurred over a much broader range of forces (Figure 5E-H). Unlike integrin *α*_5_*β*_1_ whose switching times $t_{\text{SW}\pm }$ were too brief to measure (Figure 5A), hence mimicking a digital on/off switch, the counterpart values for *α*_V_*β*_3_ were long enough to be measurable, exhibiting the characteristic of a more gradual transition. Their $\langle t_{\text{SW}\pm }\rangle$ (Figure 5E) and $\langle t_{0\pm }\rangle$ (Figure 5F) displayed similar trends. Compared to the Ca^2+^/Mg^2+^ cation condition, activating the integrin with Mn^2+^ resulted in slightly shorter $\langle t_{\text{sw}+}\rangle$ and $\langle t_{0+}\rangle$ and longer $\langle t_{\text{sw}-}\rangle$ and $\langle t_{0-}\rangle$ (Figure 5E,F), consistent with the known coupling between integrin extension and activation.^28^

Like integrin *α*_5_*β*_1_, increasing force decreased $\langle t_{0+}\rangle$ and $\langle t_{\text{sw}+}\rangle$ but increased $\langle t_{0-}\rangle$ and $\langle t_{\text{sw}-}\rangle$ of integrin *α*_V_*β*_3_ (Figure 5E,F). Quantitatively, however, the response of kinetics to force was very different. Fitting eq 3 to the data in Figure 5G returned much larger $f_{1∕2}$ values (22.67 ± 0.09 and 17.76 ± 0.14 pN in Mn^2+^ and Ca^2+^/Mg^2+^, respectively) and much smaller Δx values (0.5 ± 0.1 and 0.7 ± 0.2 nm in Mn^2+^ and Ca^2+^/Mg^2+^, respectively). These values predict that integrin *α*_V_*β*_3_ can undergo bending and unbending at a much higher force level and under a much broader range of forces, agreeing with our experimental observations. The much weaker force-dependency of integrin *α*_V_*β*_3_ bending/unbending kinetics can be seen in Figure 5H: within the force range of 4.0–28.0 pN where sufficient events were collected for statistical analysis, the population ratio $\langle t_{0-}\rangle ∕\langle t_{0+}\rangle$ of extended over bent *α*_V_*β*_3_ only increased by 8.8-fold (a force sensitivity of ~0.37-fold/pN) in Mn^2+^ and by 13-fold (a force sensitivity of ~0.54-fold/pN) in Ca^2+^/Mg^2+^, revealing a >100-fold greater resistance to force modulation than integrin *α*_5_*β*_1_. We also reanalyzed our previously published data of force-dependent integrin *α*_V_*β*_3_ bending/unbending conformational changes on cell surface,^11^ finding $f_{1∕2}$ and Δx values similar to cell-free integrin *α*_V_*β*_3_ (Supp. Table 2), indicating that these conformational changes are mainly modulated by force but not the cell environment. Together, these results demonstrated distinctive mechanisms of force modulation on integrins *α*_5_*β*_1_ and *α*_V_*β*_3_ (un)bending: a “digital” modulation for *α*_5_*β*_1_ and an “analogous” modulation for *α*_V_*β*_3_.

Interestingly, the distinctive mechanosensitivities of integrins *α*_5_*β*_1_ and *α*_V_*β*_3_ support their respective mechanosignaling roles in focal adhesion. The “digital” unbending of integrin *α*_5_*β*_1_ by force allows the cell to quickly sense extracellular stretching above a threshold, and initiate integrin *α*_5_*β*_1_ recruitment and clustering to form strong adhesion.^8^ Furthermore, around the threshold force (7.4 pN), integrin *α*_5_*β*_1_ quickly switches back-and-forth between the bent and extended conformations (>10 Hz), which could trigger fast oscillation in binding force magnitude, and therefore the strong CMR effect^36^ of integrin *α*_5_*β*_1_ to reinforce adhesion. On the other hand, the “analogous” modulation gradually shifts the conformational equilibrium of integrin *α*_V_*β*_3_ over a wide force range. This enables each integrin *α*_V_*β*_3_ molecule to act as a “ruler” for the cell to “measure” the local extracellular stretching force and matrix rigidity. As a result, when expressed on the same cell, the two integrin species can cooperate to allow the cell to both quickly adhere to the substrate and sense substrate stiffness. This cooperation may occur in and facilitate a wide range of mechanobiological processes, e.g., stem cell differentiation, angiogenesis, and bone and tumor development.^40-43^

### Explaining the Distinctive Switching Times of Integrins *α*_5_*β*_1_ and *α*_V_*β*_3_ Conformational Changes by MD Simulations.

**Figure 6.**
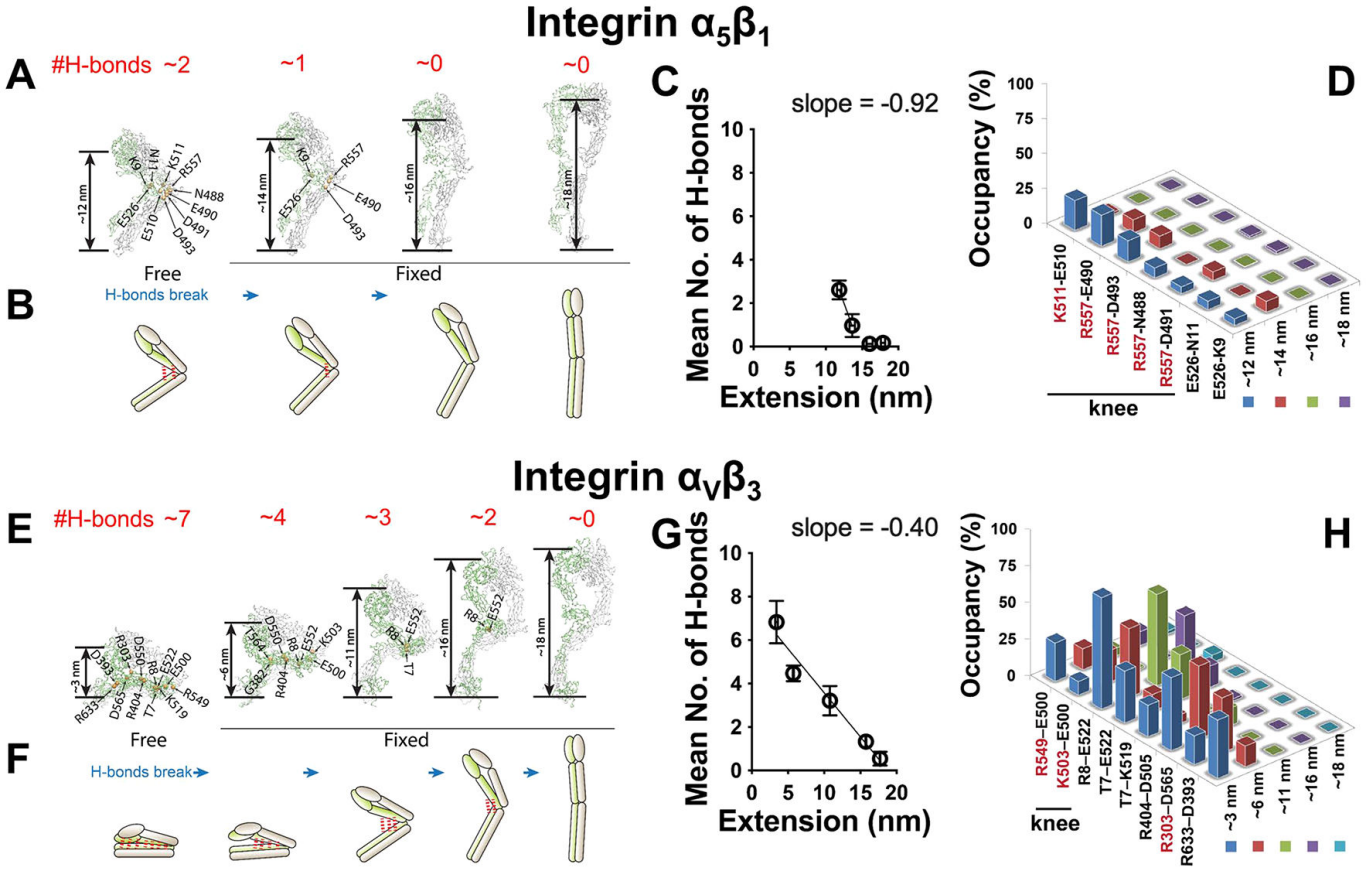
MD simulations of integrins *α*_5_*β*_1_ and *α*_V_*β*_3_ unbending conformational change. A,E. Snapshots of representative integrin *α*_5_*β*_1_ (A) and *α*_V_*β*_3_ (E) conformations (bent, 2 or 3 intermediate, and extended) observed from the MD simulations in which the most commonly observed H-bonds are indicated by their donor and acceptor residues. The red number on top of each panel represents the average number of H-bonds observed in 5 independent MD simulations. B,F. Cartoons depicting the average numbers and locations of H-bonds in relation to the head-to-tail distances during integrins *α*_5_*β*_1_ (B) and *α*_V_*β*_3_ (F) unbending. C,G. Change of average number of H-bonds (mean ± s.e.m., from 5 independent runs of MD simulations) between integrin headpiece and tailpiece during integrin *α*_5_*β*_1_ (C) and *α*_V_*β*_3_ (G) unbending. Linear fitting was applied to the first two points in (C) and all points in (G) to estimate the speed of H-bond breakage, as reflected by the slope. D,H. Average occupancy of the most frequently formed 7 H-bonds in a bent integrin *α*_5_*β*_1_ (D) or the most frequently formed 8 H-bonds in a bent *α*_V_*β*_3_ (H) when the integrin unbents to certain head-to-tail distances. Amino acids in integrin *α* and *β* chains are shown in red and black, respectively.

The orders of magnitude longer $t_{\text{sw}\pm }$ of integrin *α*_V_*β*_3_ than *α*_5_*β*_1_ is intriguing. To explain this difference, we hypothesize that *α*_V_*β*_3_ conformational changes may involve a random sequence of formation/disruption of hydrogen bonds (H-bonds) that does not occur for integrin *α*_5_*β*_1_ (refs 44, 45), resulting in a slower and more complex submolecular process for integrin *α*_V_*β*_3_ than *α*_5_*β*_1_. To test this hypothesis, we performed steered molecular dynamics (SMD) simulations on integrins *α*_5_*β*_1_ (PDB code 7NXD) and *α*_V_*β*_3_ (PDB code 3IJE) by applying external pulling forces to their headpieces. Unbending of both integrins was accompanied by the disruption of H-bonds, with larger numbers in integrin *α*_V_*β*_3_ than *α*_5_*β*_1_ (Supp. Figure 2). To acquire more quantitative information while minimizing the artifact introduced by fast force loading, we further performed free molecular dynamics (MD) simulations with integrins *α*_5_*β*_1_ and *α*_V_*β*_3_ at their bent conformation without loading or restraint. In addition, we obtained from the above SMD simulations 3–4 intermediate structures with different head-to-tail lengths, from 14 to 18 nm for *α*_5_*β*_1_ (Figure 6A) and from 6 to 18 nm for *α*_V_*β*_3_ (Figure 6E) and carried out MD simulations on these structures with the molecular length restrained. H-bonds were observed to constantly form and break between the headpiece and tailpiece of both integrins in their respective bent conformations, but the time-averaged numbers differed greatly: ~2 and ~7, respectively, for integrins *α*_5_*β*_1_ and *α*_V_*β*_3_ (Figure 6C,G). As the length of integrin *α*_5_*β*_1_ increased, its ~2 H-bonds were rapidly disrupted during the initial phase of unbending at an average rate of ~1 bond/nm of extension (Figure 6A-C). In contrast, the ~7 H-bonds in integrin *α*_V_*β*_3_ were disrupted much slower which occurred across the whole course of unbending (~0.4 bond/nm of extension) (Figure 6E-G), requiring nearly an order of magnitude longer extension to break all the H-bonds than integrin *α*_5_*β*_1_. Interestingly, in integrin *α*_5_*β*_1_ 5 out of the 7 most frequently formed H-bonds were in the integrin knee region (Figure 6D). This contrasts with integrin *α*_V_*β*_3_ where the most frequently formed 8 H-bonds were spatially equally distributed along the headpiece-tailpiece interface, and only 2 of them were in the knee region (Figure 6H). Among them, the H-bond most proximal to the integrin knee region (R8-E522) was not disrupted until the integrin reached full extension, whereas H-bonds distal to the knee region (e.g., R633-D393) were disrupted as soon as integrin *α*_V_*β*_3_ started to unbend (Figure 6H). These results indicate a direct correlation between the H-bonds’ distance to the knee and the chronological sequence of their disruption. The above observations help explain the distinctive (un)bending dynamics of the two integrins studied here and provide the rationale for the energy landscapes and transition models below.

### Constructing Energy Landscapes and Transition Models for Integrins $\alpha_{5}\beta_{1}$ and $\alpha_{V}\beta_{3}$ Conformational Changes.

**Figure 7.**
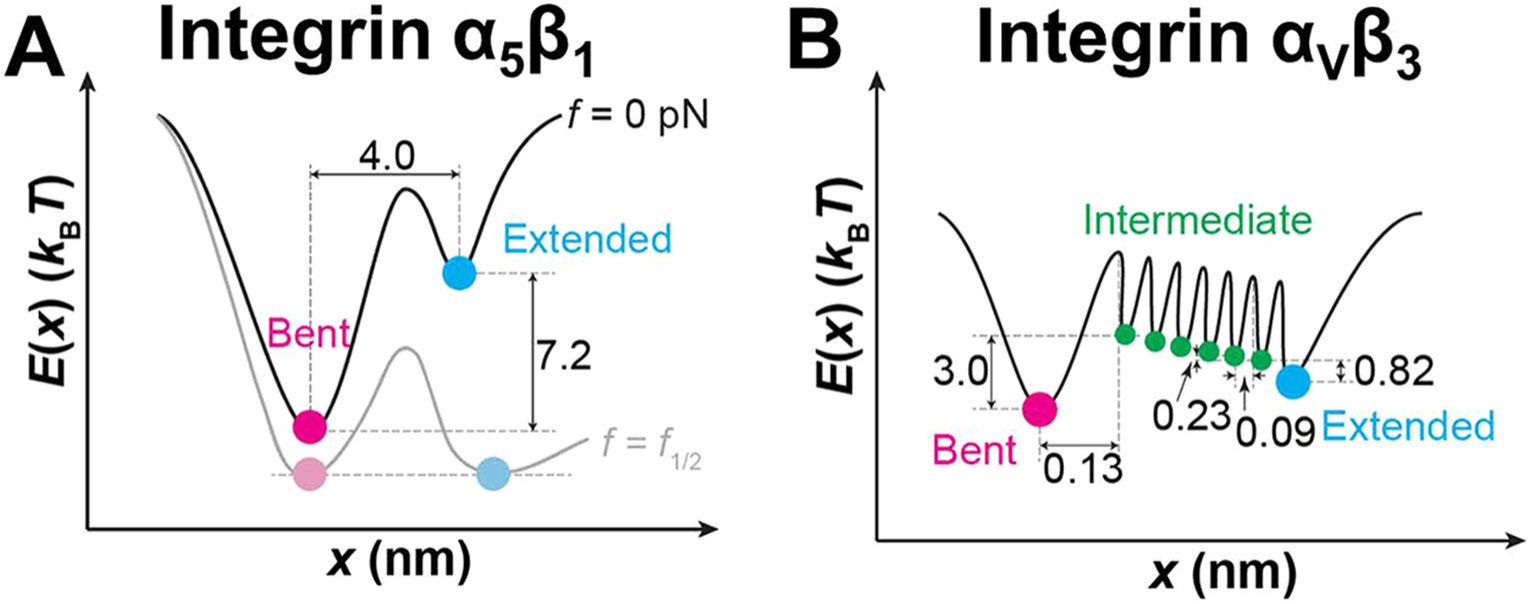
Energy landscapes of integrins *α*_5_*β*_1_ and *α*_V_*β*_3_ bending and unbending conformational changes. (A) Energy landscapes of integrin *α*_5_*β*_1_ ectodomain conformation under zero force (*dark curve*) and $f_{1∕2}$ (*light curve*) based on the experimental and model-fit parameters (Figure 5B). (B) Energy landscape of integrin *α*_V_*β*_3_ ectodomain conformation in Ca^2+^/Mg^2+^ under zero force based on the experimental and model-fit parameters (Figure 5E,F). Energy wells corresponding to the bent (*magenta*), intermediate (*green*), and extended (*cyan*) states are marked by different colors.

We wished to construct the corresponding energy landscapes and transition kinetic models for integrins $\alpha_{5}\beta_{1}$ and $\alpha_{V}\beta_{3}$, using the parameters listed in Suppl. Table 2. Noting that Δx and $k_{B}T\times \text{ln}\left(\frac{\langle t_{0+}l_{f=0}\rangle }{\langle t_{0-}l_{f=0}\rangle }\right)$ are the respective differences in the reaction coordinates and energies of the bottoms of the two energy wells for the bent and extended states at zero force, we first built an energy landscape for integrin *α*_5_*β*_1_ (Figure 7A). Without force, integrin *α*_5_*β*_1_ dominately stays in the bent conformation (Supp. Video 1). Force tilts the energy landscape such that the energy difference vanishes at $f_{1∕2}$, i.e., $\text{ln}\left(\frac{\langle t_{0+}l_{f=f_{1∕2}}\rangle }{\langle t_{0-}l_{f=f_{1∕2}}\rangle }\right)=0$. Thus, around $f_{1∕2}$ integrin *α*_5_*β*_1_ switches back and forth between the bent and extended conformations indefinitely using the energy from thermal agitations to hop over the force-tilted energy barrier separating the two states (Figure 7A, Supp. Video 2). With the force further increased, the energy well of the extended conformation is further deepened and the integrin *α*_5_*β*_1_ mainly stays in the extended conformation (Supp. Video 3).

However, such an energy landscape may not be appropriate for integrin *α*_V_*β*_3_, although its force-dependent $\langle t_{0\pm }\rangle$ data (Figure 5F) could still be fitted by the Bell model.^39^ This is because the above energy landscape with a single energy barrier corresponds to kinetics in which the integrin stays in one stable conformation for a period of time until rapidly transitioning to the other state–the top of the energy barrier corresponds to the transition state across which the molecule should spend virtually no time jumping. This agrees with the rapid transitions between the bent and extended integrin *α*_5_*β*_1_, but contradicts with the much slower conformational changes of integrin *α*_V_*β*_3_ (long $\langle t_{\text{sw}\pm }\rangle$) observed in our experiments (Figure 5E). Remembering our MD simulation where ~7 H-bonds holding the integrin in the bent conformation were sequentially disrupted over a long distance traversed by the integrin *α*_V_*β*_3_ headpiece during its unbending (Figure 6G; Supp. Figure 3E-I), we reason that the gradual formation and disruption of H-bonds must involve energy release and absorption, respectively, such that each H-bond would create an energy barrier in the energy landscape along the pathway of conformational change. Between the sequential disruptions of two successive H-bonds, integrin *α*_V_*β*_3_ would stay for some time in an energy well separated by the two energy barriers, i.e., a metastable state with intermediate energy. This is in sharp contrast to integrin *α*_5_*β*_1_ because the ~2 H-bonds between the headpiece and tailpiece of integrin *α*_5_*β*_1_ were disrupted nearly simultaneously over a much shorter distance over its unbending course (Figure 6C; Supp. Figure 3A-D), likely allowing their corresponding energy barriers to merge into one, which enables us to model its energy landscape by that depicted in Figure 7A.

We thus constructed an energy landscape model of integrin *α*_V_*β*_3_ with 7 energy barriers serially distributed between the bent and extended states, thereby creating 8 conformational states (one bent, 6 intermediate and one extended) (Figure 7B). In our cell-free system, the only energy source that drives the conformational transitions is microscopic thermal agitations from the macroscopically thermodynamically equilibrated environment. Since the purified protein may have no mechanism to regulate the directional tendency of conformational changes, the integrin that resides in any intermediate state could transition bidirectionally toward either bending or unbending regardless of the previous direction of its immediate past transition, i.e., the molecule may reversibly transition back-and-forth between any two adjacent states before jumping over the last energy barrier to one of the observed stable states (eq 4 and Supp. Equation 5), giving rise to the slow bending and unbending dynamics observed in our experiment. The switching time $\langle t_{\text{sw}\pm }\rangle$ is thus broken down into the unmeasurable times for the integrin to hop over the intermediate energy barriers and the measurable times for it to park in the intermediate energy wells before transitioning over to the next energy barrier. For the sake of simplicity, we further assumed that all energy barriers in integrin *α*_V_*β*_3_ between the intermediate states were identical in shape and evenly distributed between the bent and extended conformations, hence having identical transition rates between any two adjacent intermediate states: $k_{-}$ and $k_{+}$. The respective rates of transition from the bent or extended state to their adjacent intermediate states were designated as $k_{+}^{\text{Bent}}$ and $k_{-}^{\text{Extended}}$ respectively.

By treating the stochastic conformational changes as a Markov process in a finite state space, including bent, intermediate, and extended states, we built a master equation: $\frac{dS}{dt}=\mathrm{TS}$, where S is the vector of probabilities of the molecule to assume any of the states. T is a [N + 2]-by-[N + 2] matrix of transition rates, in which N=6 is the number of intermediate states (Supp. Equation 6). Using the probability vector solved from the master equation, we express the average time-to-transition $\langle t_{0\pm }\rangle$ and switch time $\langle t_{\text{sw}\pm }\rangle$ in terms of the kinetic rates (see Supp. Methods for details):

(4) {〈t0+〉=1−r(N+1)k+Bent(1−r)〈t0−〉=1−r−(N+1)k−Extended(1−r−1)〈tsw+〉=N−Nr−r+r(N+1)k+(1−r)2〈tsw−〉=N−Nr−1−r−1+r−(N+1)k−(1−r−1)2}

where $r=\frac{k_{-}}{k_{+}}$. Assuming that the transition between every two adjacent states follows the Bell model,^39^ all transition rates in eq 4 are regulated by force:

(5) $$k={kl}_{f=0}\;\text{exp}(f|{\Delta x}_{\pm }|∕k_{B}T)$$

where ${kl}_{f=0}$ is the value of k under zero force. $|{\Delta x}_{\pm }|$ is the distance from the bottom of the energy well of any intermediate state to the bottom of its adjacent energy well in the energy landscape that takes the positive sign for unbending and the negative sign for bending.

Using this model, we fitted the experimental $\langle t_{\text{sw}\pm }\rangle$ and $\langle t_{0\pm }\rangle$ vs force data simultaneously for both cation conditions (Figure 5E,F,H), showing good agreement. Fitting returned two sets of best-fit parameters, one for each cation condition, which allowed us to evaluate the parameters of the energy landscape, including differences between neighboring states: ${\Delta x}_{n}={\Delta x}_{-}^{n}+{\Delta x}_{+}^{n}$, ${\Delta G}_{n}=k_{B}T\times \text{ln}\;(k_{-}^{n+1}∕k_{+}^{n})\;(n=0\;(\text{Bent}),\;1,2,\ldots \;6)\;(‘‘n+1\;7’’$ represents “Extended” state), and plot the energy landscape of *α*_V_*β*_3_ conformational changes (Supp. Table 3, Figure 7B). As a sanity check, for both Ca^2+^/Mg^2+^ and Mn^2+^ cases we calculate the sum of these parameters, finding $\Delta x={\Delta x}_{\text{Extended}}+{\Delta x}_{\text{Bent}}+\sum_{1}^{5}{\Delta x}_{n}=0.69$ and 0.64 nm and $\Delta G=k_{B}T(\text{ln}(k_{-}^{1}∕k_{+}^{\text{Bent}})+\sum_{1}^{5}\text{ln}(k_{-}^{n+1}∕k_{+}^{n})$ 1.03 and 0.41 $k_{B}T$, $+\;\text{ln}(k_{-}^{\text{Extended}}∕k_{+}^{6})=$ corresponding to $f_{1∕2}\;=\;\Delta G∕\Delta x\;=\;6.14$ and 2.64 pN, respectively, which are comparable to the values listed in Supp. Table 2, validating that the serial energy barrier model is equivalent to the single energy barrier model in terms of both energetics and force-dependency.

To further validate our model, we used Monte Carlo simulations to perform “mock runs” based on this energy landscape, which was able to recreate integrin spontaneous bending and unbending conformational changes over time (Supp. Figure 4A,B, Supp. Videos 4-6). Our multistate model predicts that integrin may jump back-and-forth between adjacent states. Indeed, we observed that integrins occasionally paused in the middle of a bending process and reversed the course to unbend in both Monte Carlo simulations (Supp. Figure 4A, Supp. Video 4) and BFP experiments (e.g., Supp. Figure 4C). These results validate our proposed energy landscape of integrin *α*_V_*β*_3_ conformational changes. With force applied to integrin *α*_V_*β*_3_, the energy landscape is tilted and the integrin is shifted toward the extended state (Supp. Video 5, 6)

Although it is still not clear how integrin *α*_V_*β*_3_ conformational changes can persist under force conditions that are energetically unfavorable, our model seems to suggest a facilitating mechanism: the sequential formation and disruption of H-bonds serve as “stairs” for integrin *α*_V_*β*_3_ to temporarily “rest” as it moves up- and down-stairs, so that the energy differential required in each “step” is reduced. Meanwhile, we would like to point out that the model still has limitations. For instance, it assumes a one-dimensional reaction coordinate and that all energy barriers are identical in shape and evenly distributed, and considers only H-bonds but not other types of noncovalent interactions (e.g., salt bridge, hydrophobic interaction) and covalent bond reactions (e.g., thiol–disulfide exchange), which may be addressed in future studies.

## CONCLUSIONS

Force-modulated integrin bending and unbending conformational changes have previously been observed on cell surfaces.^11,12^ Here, we provide real-time single-molecule experimental data to show that purified integrin ectodomains are capable of undergoing force-modulated bending and unbending conformational changes independent of the cellular environment. Our results reveal very different biophysical characteristics for the two focal adhesion integrins: *α*_5_*β*_1_ and *α*_V_*β*_3_. The conformational changes of integrin *α*_V_*β*_3_ are more gradually modulated by force in an “analogous” fashion as opposed to the “digital” fashion seen in the integrin *α*_5_*β*_1_ case. It is reasonable to speculate that differences in mechanosensitivity generally exist across all integrin species, which directly affects how different integrins interpret and react to the biomechanical environment. Accordingly, different biomechanical features (e.g., elasticity, viscosity, and surface fluidity) probably should be adopted when designing therapeutical nanoparticles and nanomaterials that target distinctive integrin species to achieve optimal accommodation and avoid undesired cell mechanosignaling.

Among the many macromolecular systems that were found to possess the capability of force-modulated reversible conformational transitions,^46-51^ integrin *α*_V_*β*_3_ appears to be the only one identified so far that is capable of slow-kinetic sizable spontaneous conformational changes under a wide range of force without external energy source. We cannot help to speculate that more molecules with a similar attribute exist and await to be discovered. Studying these mechanosensitive structures will help us understand how they accumulate and convert small-scale thermal energy into the work required for large-scale molecular conformational changes against force. A nanoscopic module that can fulfill such a task should be of potential use in biomaterial-based nanorobots for certain movement tasks (e.g., “switch” and “hinge” movement).^52^ In this context, the present work provided not only an actual example but also a critical concept and useful design principles for the engineering of protein biomechanical machines in the field of bionanotechnology.^53^

## METHODS/EXPERIMENTAL

### Proteins, antibodies and reagents.

Previously described^16,32^ recombinant *α*_5_*β*_1_-Fc and tr*α*_5_*β*_1_-Fc were generous gifts of Martin J. Humphries (University of Manchester, UK),^54^ *α*_V_*β*_3_-Hexa-His was a kind gift of Junichi Takagi (Osaka University, Japan).^28^ *α*_5_*β*_1_-Poly-His was purchased from Sino Biological (Wayne, PA). FN biotinylated at the N-terminus was a kind gift of Andres J. Garcia (Georgia Institute of Technology, USA).^55^ The anti-FN mAb (HFN7.1) was from Developmental Studies Hybridoma Bank (Iowa City, IA). The antihuman Fc capturing mAb (GG-7) was from Sigma-Aldrich (St. Louis, MO). LIBS-2 and BMC5 was purchased from EMD Millipore (Billerica, MA). Anti-Penta-His antibody was purchased from Qiagen (Germany).

MAL-PEG3500-NHS and Biotin-PEG3500-NHS were from JenKem (Plano, TX). Nystatin, streptavidin-maleimide, and BSA were from Sigma-Aldrich. Borosilicate glass beads were from Distrilab Particle Technology (RC Leusden, The Netherlands).

### AFM Setup, Preparation, and Experiment.

Our AFM was built and calibrated in-house.^16^ A Petri-dish was directly mounted onto a piezo (P-363, Physik Instrumente, Karlsrube Germany), which was controlled by a computer program (Labview, National Instruments) with a subnanometer spatial resolution through capacitive sensor feedback. A laser (Oz Optics, Ontario, Canada) was focused on the back of the cantilever (TM microscopes, Sunnyvale, CA) end, and deflected onto a photodiode (Hamamatsu, Bridgewater, NJ) to allow the cantilever deflection to be converted to force based on the cantilever spring constant.^56^ To engage the integrins with FN, cantilever tips were incubated with 10–20 *μ*g/mL FN overnight at 4 °C, rinsed, and incubated in Tris-buffered saline (50 mM Tris-Cl, 150 mM NaCl, pH 7.5) containing 1% bovine serine albumin (BSA) for 15 min at room temperature to block nonspecific binding.^16^ For integrin coating, anti-Penta-His antibody was adsorbed on the Petri-dish, rinsed, and then incubated with 10 *μ*g/mL *α*_V_*β*_3_-Hexa-His, or GG-7 was adsorbed on the Petri-dish, rinsed, and incubated with 10 *μ*g/mL *α*_5_*β*_1_-Fc or tr*α*_5_*β*_1_-Fc for 30 min. Control experiments have been performed in a previous work (using the same instrumental setup and molecular systems)^16^ and in the present work, which ensured that the detected binding events were mostly mediated by specific binding between the integrins and FN, while nonspecific binding events were negligible.^16^

Some of the AFM experiment procedures have been described previously.^16,36^ Briefly, the Petri-dish was added with a buffer of the desired cation composition. The piezo brought the Petri-dish to contact the cantilever tip, retracted slightly and held the Petri-dish close to the tip for 0.5 s to allow bond formation, and then retracted it at a speed of 200 nm/s. The presence of an adhesion event was reflected by a positive force signal in the force-time curves. The coating of the Petri-dish was titrated to keep adhesion infrequent (<20%), a necessary condition for most of the adhesion events (>89%) to be mediated by single bonds.^22^ For force-induced unbending and rebending measurements, the Petri-dish was driven at a constant speed (200 nm/s) to load the bond to ~20 pN and retract at the same speed to unload the bond. The (un)bending events were identified and parameters measured from the force-time traces (cf. Figure 1B). For CMR measurements, the Petri-dish was driven to move cyclically so the integrin─FN bond underwent force loading and unloading and then held at a preset force (cf. Figure 4A,C).^36^ Lifetime was measured from the instant when the force reached the desired level to the instant of bond dissociation. The collected lifetime data were categorized into bins of successive force ranges, and averaged within each force bin to plot the lifetime curve. For force-ramp after a cyclic loading–unloading cycle with a high peak force, the piezo was retracted at a very low speed (1 nm/s) to allow observation of repetitive unbending and bending events over a prolonged period until bond rupture.

### RBC and Glass Bead Preparation.

Human blood (8–10 *μ*L) was obtained from finger prick following a protocol approved by the Institutional Review Board of Georgia Institute of Technology (protocol number H12354) and The University of Texas Medical Branch (protocol number 22-0015). RBCs were isolated and biotinylated by incubating with Biotin-PEG3500-NHS solution.^11^ The biotinylated RBCs were then incubated with nystatin, which would swell the RBCs to near spherical shapes.

The procedure for bead functionalization has been described.^57^ Briefly, after thiolation, glass beads were incubated with streptavidin-maleimide, anti-Penta-His antibody cross-linked with MAL-PEG3500-NHS, or LIBS-2 cross-linked with MAL-PEG3500-NHS overnight. Streptavidin-coated beads were incubated with biotinylated FN solution for 2 h. Anti-Penta-His antibody coated beads were incubated with *α*_V_*β*_3_-Hexa-His or *α*_5_*β*_1_-Poly-His solution for 3 h. LIBS-2 coated beads were used without further incubation. All beads after incubation were washed with and resuspended in phosphate buffer (27.6 g/L NaH_2_PO_4_·H_2_O, 28.4 g/L Na_2_HPO_4_).

### Platelet Isolation.

The procedure for collecting human venous blood was approved by the Institutional Review Board of the Georgia Institute of Technology (protocol number H12354). Blood was collected from healthy volunteers into tubes containing anticoagulant and activation-suppressing agents, and centrifuged at 200*g* for 15 min to isolate platelet rich plasma, which was centrifuged at 900*g* for another 10 min to isolate the platelet pellet. The platelet pellet was resuspended in a platelet washing buffer (4.3 mM K_2_HPO_4_, 4.3 mM Na_2_HPO_4_, 24.3 mM NaH_2_PO_4_, 113 mM NaCl, 5.5 mM d-glucose, 10 mM theophylline, 20 U/mL clexane, 0.01 U/mL apyrase, 1% BSA, pH 6.5), rested for 15 min, and centrifuged again. Finally, the platelet pellet was resuspended into a HEPES-Tyrode buffer (134 mM NaCl, 12 mM NaHCO_3_, 2.9 mM KCl, 0.34 mM sodium phosphate monobasic, 5 mM HEPES, and 5 mM glucose, 0.02 U/mL apyrase, 1% BSA, pH 7.4) ready for experiments.

### BFP Setup, Preparation, and Experiment.

Our BFP apparatus has been described previously.^12,57^ A chamber mounted on an inverted microscope (Nikon TiE, Nikon) was filled with an experimental buffer supplemented with 1% BSA to block nonspecific binding and cations (1 mM Ca^2+^/Mg^2+^ or 2 mM Mn^2+^). A biotinylated RBC was aspirated by a micropipette to act as a force transducer (Figures 2A and 3E, *left*), the spring constant of which was set to 0.5 pN/nm when assessing integrin *α*_5_*β*_1_, and to 0.25 or 0.3 pN/nm when assessing integrin *α*_V_*β*_3_.^11^ A probe bead bearing FN or LIBS-2 was attached to the apex of the RBC via streptavidin–biotin interaction. An integrin *α*_V_*β*_3_-functionalized bead or a platelet was aspirated by an opposing micropipette (Figures 2A and 3E, *right*) as the target, and driven by a piezoelectric translator (Physical Instrument) to repeatedly touch with the probe bead and retract. The probe bead’s position was tracked by a high-speed camera.

The BFP measurement procedures for bond lifetime, (un)bending, and CMR are similar to those for AFM experiments, wherein a tensile force signal indicated an adhesion event between the probe bead and the target. FN coating on the probe bead was titrated to maintain infrequent adhesion (<20%).^22^ For integrin *α*_5_*β*_1_ experiments, all adhesion bonds were ramped until they broke. For integrin *α*_V_*β*_3_ experiments, upon the detection of an adhesion event, the target pipet was held at a desired position (reflected by the initial clamping force) to wait for the bond to dissociate.

### Molecular Stiffness Measurement.

As previously described,^11,12^ force vs time data from AFM and BFP experiments were transformed to “force vs. extension” data (cf. from Figure 1B to 1E). The tensile force portion of the “force vs. extension” data was fitted by a line and the slope was taken as the stiffness of the integrin-FN complex. The value mainly reflects the integrin stiffness as the contribution from FN is negligible.

### Molecular Dynamics (MD) Simulations of Integrins *α*_5_*β*_1_ and *α*_V_*β*_3_.

The ectodomain crystal structure of integrins *α*_5_*β*_1_ (PDB code 7NXD)^21^ and *α*_V_*β*_3_ (PDB code 3IJE)^58^ was used to perform the MD simulation with GROMACS.^59^ The TIP3P model was used to depict water molecules. Na^+^ and Cl^−^ were added to neutralize the system and maintain the physiological salt condition (150 mM). The CHARM36 force field^60^ was used to describe the interactions of the protein and the solvent. CHARMM Additive All-Atom Force Field^61^ was used to describe the sugar. Simulations began with minimizing the energy of the protein using steep decent methods, and then the system temperature was raised from 3 to 300 K in an annealing simulation with controlled volume within 500 ps, followed by another 500 ps simulation in NVT ensemble. Afterward, a 1-ns simulation was performed in an NPT ensemble at 300 K and 1 atm. The temperature and pressure were controlled by a v-rescale thermostat and Parrinello–Rahman barostat, respectively.^62^ In the annealing, NVT, and NPT simulations, the positions of the heavy atoms of the integrin were restrained.

In the steered molecular dynamics (SMD) simulation, the C-terminal C*α* atom of both *α* and *β* tail was restrained, and a group of atoms in the integrin head (C*α* of residues 113–117, 151–156, 190–197, 244–250, 306-310, and 329–332 of *β*_3_ subunit for *α*_V_*β*_3_ and 124–130, 161–167, 199–206, 252–258, 312–317, 336–339 of *β*_1_ subunit for *α*_5_*β*_1_) were pulled at a speed of 0.5 nm/ns. Five independent pulling simulations were performed for both *α*_V_*β*_3_ and for *α*_5_*β*_1_. From these simulation trajectories, the structures of three partially extended (~6 nm, ~11 nm, ~16 nm) and a fully extended (~18 nm) *α*_V_*β*_3_ integrins and two partially extended (~14 nm, ~16 nm) and a fully extended (~18 nm) *α*_5_*β*_1_ integrins were obtained. These structures were further used in the MD simulations.

In the MD simulations of the partially and fully extended integrin structures acquired from the above SMD simulations, the C-terminal C*α* atom of *β* tail was restrained, and the clamping force was applied to the same group of atoms as in the SMD simulation, with the pulling speed set to 0. On the other hand, for MD simulation of the bent *α*_5_*β*_1_ and *α*_V_*β*_3_ integrins, no restraint was applied. The numbers of H-bonds in all the above bent, partially extended, and fully extended integrin structures were analyzed with a threshold distance of 0.3 nm and a donor–acceptor angle of 20°.

### Statistical Analysis.

Statistical significance was assessed by unpaired or paired, two-tailed Student’s *t* test or one-way ANOVA.

## Supplementary Material

Supp. Video 1 Supplementary Video 1. Monte Carlo simulated transitions between the bent and extended conformations of integrin *α*_5_*β*_1_ in the absence of externally applied force (MP4)

Supp. Video 3 Supplementary Video 3. Monte Carlo simulated transitions between the bent and extended conformations of integrin *α*_5_*β*_1_ under 15 pN of pulling force (MP4)

Supp. Video 2 Supplementary Video 2. Monte Carlo simulated transitions between the bent and extended conformations of integrin *α*_5_*β*_1_ under 7.4 pN of pulling force (MP4)

Supp. Video 4 Supplementary Video 4. Monte Carlo simulated transitions between the bent, intermediate, and extended conformations of integrin *α*_V_*β*_3_ in the absence of externally applied force (MP4)

Supp. Video 6 Supplementary Video 6. Monte Carlo simulated transitions between the bent, intermediate, and extended conformations of integrin *α*_V_*β*_3_ under 12 pN of pulling force (MP4)

Supp. Video 5 Supplementary Video 5. Monte Carlo simulated transitions between the bent, intermediate, and extended conformations of integrin *α*_V_*β*_3_ under 6 pN of pulling force (MP4)

Supp Information

## References

[1] Sun Z; Guo SS; Fassler R Integrin-mediated mechanotransduction. J. Cell Biol 2016, 215, 445–456. 27872252 10.1083/jcb.201609037 PMC5119943

[2] Winograd-Katz SE; Fassler R; Geiger B; Legate KR The integrin adhesome: from genes and proteins to human disease. Nature reviews. Molecular Cell Biology 2014, 15, 273–288. 24651544 10.1038/nrm3769

[3] Dhavalikar P.; Review of Integrin-Targeting Biomaterials in Tissue Engineering. Adv. Healthc Mater 2020, 9, No. e2000795. 10.1002/adhm.202000795 PMC7960574 32940020

[4] Montet X; Montet-Abou K; Reynolds F; Weissleder R; Josephson L Nanoparticle imaging of integrins on tumor cells. Neoplasia 2006, 8, 214–222. 16611415 10.1593/neo.05769 PMC1578521

[5] Wu PH; Opadele AE; Onodera Y; Nam JM Targeting Integrins in Cancer Nanomedicine: Applications in Cancer Diagnosis and Therapy. Cancers 2019, 11, 1783. 31766201 10.3390/cancers11111783 PMC6895796

[6] Guo P.; Nanoparticle elasticity directs tumor uptake. Nat. Commun 2018, 9, 130. 29317633 10.1038/s41467-017-02588-9 PMC5760638

[7] Anselmo AC; Elasticity of nanoparticles influences their blood circulation, phagocytosis, endocytosis, and targeting. ACS Nano 2015, 9, 3169–3177. 25715979 10.1021/acsnano.5b00147

[8] Roca-Cusachs P; Gauthier NC; Del Rio A; Sheetz MP Clustering of alpha(5)beta(1) integrins determines adhesion strength whereas alpha(v)beta(3) and talin enable mechanotransduction. Proc. Natl. Acad. Sci. U.S.A 2009, 106, 16245–16250. 19805288 10.1073/pnas.0902818106 PMC2752568

[9] Rossier O; Octeau V; Sibarita J-B; Leduc C; Tessier B; Nair D; Gatterdam V; Destaing O; Albiges-Rizo C; Tampe R; Cognet L; Choquet D; Lounis B; Giannone G Integrins beta(1) and beta(3) exhibit distinct dynamic nanoscale organizations inside focal adhesions. Nat. Cell Biol 2012, 14, 1057–1067. 23023225 10.1038/ncb2588

[10] Danen EH; Sonneveld P; Brakebusch C; Fassler R; Sonnenberg A The fibronectin-binding integrins alpha5beta1 and alphavbeta3 differentially modulate RhoA-GTP loading, organization of cell matrix adhesions, and fibronectin fibrillogenesis. J. Cell Biol 2002, 159, 1071–1086. 12486108 10.1083/jcb.200205014 PMC2173988

[11] Chen Y; Lee H; Tong H; Schwartz M; Zhu C Force regulated conformational change of integrin alphaVbeta3. Matrix Biology 2017, 60–61, 70–85. 10.1016/j.matbio.2016.07.002 PMC5237428 27423389

[12] Chen W; Lou J; Evans EA; Zhu C Observing force-regulated conformational changes and ligand dissociation from a single integrin on cells. J. Cell Biol 2012, 199, 497–512. 23109670 10.1083/jcb.201201091 PMC3483124

[13] Wong JY; Kuhl TL; Israelachvili JN; Mullah N; Zalipsky S Direct measurement of a tethered ligand-receptor interaction potential. Science 1997, 275, 820–822. 9012346 10.1126/science.275.5301.820

[14] Chen Y; Ju L; Rushdi M; Ge C; Zhu C Receptor-mediated cell mechanosensing. Molecular Biology of the Cell 2017, 28, 3134–3155. 28954860 10.1091/mbc.E17-04-0228 PMC5687017

[15] Magnusson MK; Mosher DF Fibronectin: structure, assembly, and cardiovascular implications. Arterioscler., Thromb., Vasc. Biol 1998, 18, 1363–1370. 9743223 10.1161/01.atv.18.9.1363

[16] Kong F; Garcia AJ; Mould AP; Humphries MJ; Zhu C Demonstration of catch bonds between an integrin and its ligand. J. Cell Biol 2009, 185, 1275–1284. 19564406 10.1083/jcb.200810002 PMC2712956

[17] Ying J; Ling Y; Westfield LA; Sadler JE; Shao JY Unfolding the A2 domain of von Willebrand factor with the optical trap. Biophysical Journal 2010, 98, 1685–1693. 20409490 10.1016/j.bpj.2009.12.4324 PMC2856187

[18] Chen Y.; An integrin alphaIIbbeta3 intermediate affinity state mediates biomechanical platelet aggregation. Nature Materials 2019, 18, 760–769. 30911119 10.1038/s41563-019-0323-6 PMC6586518

[19] Li J; Springer TA Integrin extension enables ultrasensitive regulation by cytoskeletal force. Proc. Natl. Acad. Sci. U.S.A 2017, 114, 4685–4690. 28416675 10.1073/pnas.1704171114 PMC5422820

[20] Cormier A.; Cryo-EM structure of the alphavbeta8 integrin reveals a mechanism for stabilizing integrin extension. Nat. Struct Mol. Biol 2018, 25, 698–704. 30061598 10.1038/s41594-018-0093-x PMC6214843

[21] Schumacher S.; Structural insights into integrin alpha(5)beta(1) opening by fibronectin ligand. Sci. Adv 2021, 7 (19), DOI: 10.1126/sciadv.abe9716. PMC8104898 33962943

[22] Chesla SE; Selvaraj P; Zhu C Measuring two-dimensional receptor-ligand binding kinetics by micropipette. Biophysical journal 1998, 75, 1553–1572. 9726957 10.1016/S0006-3495(98)74074-3 PMC1299830

[23] Yao M.; The mechanical response of talin. Nat. Commun 2016, 7, 11966. 27384267 10.1038/ncomms11966 PMC4941051

[24] Du X.; Long range propagation of conformational changes in integrin alpha IIb beta 3. J. Biol. Chem 1993, 268, 23087–23092. 7693683

[25] Frelinger AL 3rd; Du XP; Plow EF; Ginsberg MH Monoclonal antibodies to ligand-occupied conformers of integrin alpha IIb beta 3 (glycoprotein IIb-IIIa) alter receptor affinity, specificity, and function. J. Biol. Chem 1991, 266, 17106–17111. 1894607

[26] Chen W.; Molecular Dynamics Simulations of Forced Unbending of Integrin *α*V*β*3. PLoS Comp Biol. 2011, 7, No. e1001086. 10.1371/journal.pcbi.1001086 PMC3040657 21379327

[27] Xiong JP; Crystal structure of the extracellular segment of integrin alpha Vbeta3. Science 2001, 294, 339–345. 11546839 10.1126/science.1064535 PMC2885948

[28] Takagi J.; Petre BM; Walz T; Springer TA Global conformational rearrangements in integrin extracellular domains in outside-in and inside-out signaling. Cell 2002, 110, 599–511. 12230977 10.1016/s0092-8674(02)00935-2

[29] Miyamoto S; Akiyama SK; Yamada KM Synergistic roles for receptor occupancy and aggregation in integrin transmembrane function. Science 1995, 267, 883–885. 7846531 10.1126/science.7846531

[30] Yamada KM; Miyamoto S Integrin transmembrane signaling and cytoskeletal control. Curr. Opin. Cell Biol 1995, 7, 681–689. 8573343 10.1016/0955-0674(95)80110-3

[31] Hynes RO Integrins: bidirectional, allosteric signaling machines. Cell 2002, 110, 673–687. 12297042 10.1016/s0092-8674(02)00971-6

[32] Elosegui-Artola A; Mechanical regulation of a molecular clutch defines force transmission and transduction in response to matrix rigidity. Nat. Cell Biol 2016, 18, 540–548. 27065098 10.1038/ncb3336

[33] Kulke M; Langel W Molecular dynamics simulations to the bidirectional adhesion signaling pathway of integrin alphaV beta3. Proteins 2020, 88, 679–688. 31693219 10.1002/prot.25849

[34] Kim J; Topological Adaptation of Transmembrane Domains to the Force-Modulated Lipid Bilayer Is a Basis of Sensing Mechanical Force. Current Biology 2020, 30, 1614–1625. 32169208 10.1016/j.cub.2020.02.028 PMC7202955

[35] Changede R; Cai H; Wind SJ; Sheetz MP Integrin nanoclusters can bridge thin matrix fibres to form cell-matrix adhesions. Nature Materials 2019, 18, 1366–1375. 31477904 10.1038/s41563-019-0460-y PMC7455205

[36] Kong F.; Cyclic mechanical reinforcement of integrin-ligand interactions. Molecular Cell 2013, 49, 1060–1068. 23416109 10.1016/j.molcel.2013.01.015 PMC3615084

[37] Lee H; Eskin SG; Ono S; Zhu C; McIntire LV Force-history dependence and cyclic mechanical reinforcement of actin filaments at the single molecular level. J. Cell Sci 2019, 132 (4), jcs216911 DOI: 10.1242/jcs.216911. 30659118 PMC6398476

[38] Dembo M; Torney DC; Saxman K; Hammer D The reaction-limited kinetics of membrane-to-surface adhesion and detachment. Proc. R. Soc. London B Biol. Sci 1988, 234, 55–83. 2901109 10.1098/rspb.1988.0038

[39] Bell GI Models for the specific adhesion of cells to cells. Science 1978, 200, 618–627. 347575 10.1126/science.347575

[40] Geoghegan IP; Hoey DA; McNamara LM Integrins in Osteocyte Biology and Mechanotransduction. Curr. Osteoporos Rep 2019, 17, 195–206. 31250372 10.1007/s11914-019-00520-2

[41] Flournoy J; Ashkanani S; Chen Y Mechanical regulation of signal transduction in angiogenesis. Frontiers in Cell and Developmental Biology 2022, 10, No. 933474. 36081909 10.3389/fcell.2022.933474 PMC9447863

[42] Yang B.; Stopping transformed cancer cell growth by rigidity sensing. Nature Materials 2020, 19, 239–250. 31659296 10.1038/s41563-019-0507-0 PMC7477912

[43] Murphy WL; McDevitt TC; Engler AJ Materials as stem cell regulators. Nature Materials 2014, 13, 547–557. 24845994 10.1038/nmat3937 PMC4163547

[44] Lin F-Y; Zhu J; Eng ET; Hudson NE; Springer TA *β*-Subunit binding is sufficient for ligands to open the integrin *α*IIb*β*3 headpiece. J. Biol. Chem 2016, 291, 4537–4546. 26631735 10.1074/jbc.M115.705624 PMC4813479

[45] Zhu J.; Structure of a complete integrin ectodomain in a physiologic resting state and activation and deactivation by applied forces. Molecular Cell 2008, 32, 849–861. 19111664 10.1016/j.molcel.2008.11.018 PMC2758073

[46] Das DK; Pre-T Cell Receptors (Pre-TCRs) Leverage Vbeta Complementarity Determining Regions (CDRs) and Hydrophobic Patch in Mechanosensing Thymic Self-ligands. J. Biol. Chem 2016, 291, 25292–25305. 27707880 10.1074/jbc.M116.752865 PMC5207233

[47] Zhang XF; Zhang W; Quach ME; Deng W; Li R Force-Regulated Refolding of the Mechanosensory Domain in the Platelet Glycoprotein Ib-IX Complex. Biophysical Journal 2019, 116, 1960–1969. 31030883 10.1016/j.bpj.2019.03.037 PMC6531785

[48] Popa I.; A HaloTag Anchored Ruler for Week-Long Studies of Protein Dynamics. J. Am. Chem. Soc 2016, 138, 10546–10553. 27409974 10.1021/jacs.6b05429 PMC5510598

[49] Eckels EC; Haldar S; Tapia-Rojo R; Rivas-Pardo JA; Fernandez JM The Mechanical Power of Titin Folding. Cell Rep. 2019, 27, 1836–1847. 31067467 10.1016/j.celrep.2019.04.046 PMC6937205

[50] Tapia-Rojo R; Eckels EC; Fernandez JM Ephemeral states in protein folding under force captured with a magnetic tweezers design. Proc. Natl. Acad. Sci. U.S.A 2019, 116, 7873–7878. 30936303 10.1073/pnas.1821284116 PMC6475431

[51] Wu P.; Mechano-regulation of Peptide-MHC Class I Conformations Determines TCR Antigen Recognition. Molecular Cell 2019, 73, 1015. 30711376 10.1016/j.molcel.2018.12.018 PMC6408234

[52] Wang B; Kostarelos K; Nelson BJ; Zhang L Trends in Micro-/Nanorobotics: Materials Development, Actuation, Localization, and System Integration for Biomedical Applications. Advanced Materials 2021, 33, No. e2002047. 33617105 10.1002/adma.202002047

[53] Soto F; Wang J; Ahmed R; Demirci U Medical Micro/Nanorobots in Precision Medicine. Advanced Science 2020, 7, 2002203. 33173743 10.1002/advs.202002203 PMC7610261

[54] Coe AP; Generation of a minimal alpha5beta1 integrin-Fc fragment. J. Biol. Chem 2001, 276, 35854–35866. 11389148 10.1074/jbc.M103639200

[55] Petrie TA; Capadona JR; Reyes CD; Garcia AJ Integrin specificity and enhanced cellular activities associated with surfaces presenting a recombinant fibronectin fragment compared to RGD supports. Biomaterials 2006, 27, 5459–5470. 16846640 10.1016/j.biomaterials.2006.06.027

[56] Hutter JL; Bechhoefer J Calibration of atomic-force microscope tips. Rev. Sci. Instrum 1993, 64, 1868–1873.

[57] Chen Y.; Fluorescence Biomembrane Force Probe: Concurrent Quantitation of Receptor-ligand Kinetics and Binding-induced Intracellular Signaling on a Single Cell. J. Visualized Exp 2015, No. e52975. 10.3791/52975 PMC4544851 26274371

[58] Xiong JP; Crystal structure of the complete integrin alphaVbeta3 ectodomain plus an alpha/beta transmembrane fragment. J. Cell Biol 2009, 186, 589–600. 19704023 10.1083/jcb.200905085 PMC2733745

[59] Van Der Spoel D; GROMACS: fast, flexible, and free. Journal of Computational Chemistry 2005, 26, 1701–1718. 16211538 10.1002/jcc.20291

[60] Huang J; MacKerell AD Jr. CHARMM36 all-atom additive protein force field: validation based on comparison to NMR data. Journal of Computational Chemistry 2013, 34, 2135–2145. 23832629 10.1002/jcc.23354 PMC3800559

[61] Raman EP; Guvench O; MacKerell AD Jr. CHARMM additive all-atom force field for glycosidic linkages in carbohydrates involving furanoses. Journal of Physical Chemistry. B 2010, 114, 12981–12994. 20845956 10.1021/jp105758h PMC2958709

[62] Bussi G; Donadio D; Parrinello M Canonical sampling through velocity rescaling. J. Chem. Phys 2007, 126, No. 014101. 17212484 10.1063/1.2408420

