# Supplementary material for: Force-Regulated Spontaneous Conformational Changes of Integrins α5β1 and αVβ3
Source: ACS Nano. Author manuscript; Available in PMC 2024 Feb 16. (PMC10786158; doi:10.1021/acsnano.3c06253)
Supplement: Supp Information [file NIHMS1953748-supplement-Supp_Information.pdf]

Supporting Information

Force-regulated spontaneous conformational changes of integrins $\alpha_5\beta_1$ and $\alpha_V\beta_3$

Yunfeng Chen[1,2,3†*], Zhenhai Li[4†*], Fang Kong[1,2,5,6†], Lining Arnold Ju[2,5,7,8], and Cheng Zhu[1,2,5*]

[1]Woodruff School of Mechanical Engineering and [2]Petit Institute for Bioengineering and Biosciences, Georgia Institute of Technology, Atlanta, Georgia 30332, USA
[3]Department of Biochemistry and Molecular Biology and Department of Pathology, The University of Texas Medical Branch, Galveston, Texas 77555, USA
[4]Shanghai Key Laboratory of Mechanics in Energy Engineering, Shanghai Institute of Applied Mathematics and Mechanics, School of Mechanics and Engineering Science, Shanghai University, Shanghai 200072, China
[5]Coulter Department of Biomedical Engineering, Georgia Institute of Technology, Atlanta, Georgia 30332, USA
[6]School of Biological Science, Nanyang Technological University, Singapore 637551, Singapore
[7]School of Biomedical Engineering, The University of Sydney, Darlington, NSW 2008, Australia
[8]Charles Perkins Centre, The University of Sydney, Camperdown, NSW 2006, Australia

[†]These authors contributed equally.
*Correspondence to Yunfeng Chen (yunfchen@utmb.edu), Zhenhai Li (lizhshu@shu.edu.cn) and Cheng Zhu (cheng.zhu@bme.gatech.edu)

Supplementary Figures

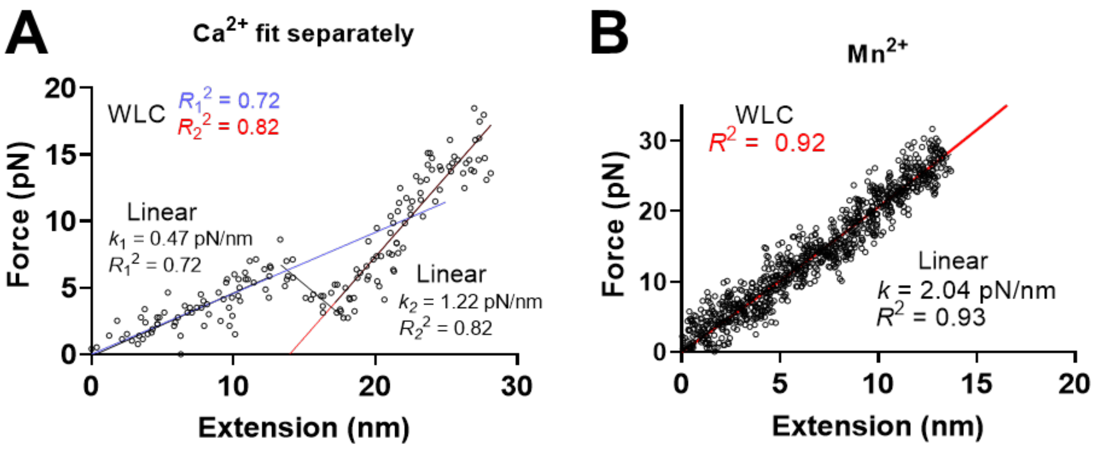

Supplementary figure 1. Fitting the force vs. extension curves of integrin $\alpha_5\beta_1$ with Worm-Like Chain (WLC) model and linear model. (A) Separately fitting the pre- and post-unbending segments of an unbending event containing force vs. extension curve (in Ca^{2+} condition) using WLC and linear models. (B) Fitting a force vs. extension curve containing no unbending event (in Mn^{2+} condition) with linear and WLC models.

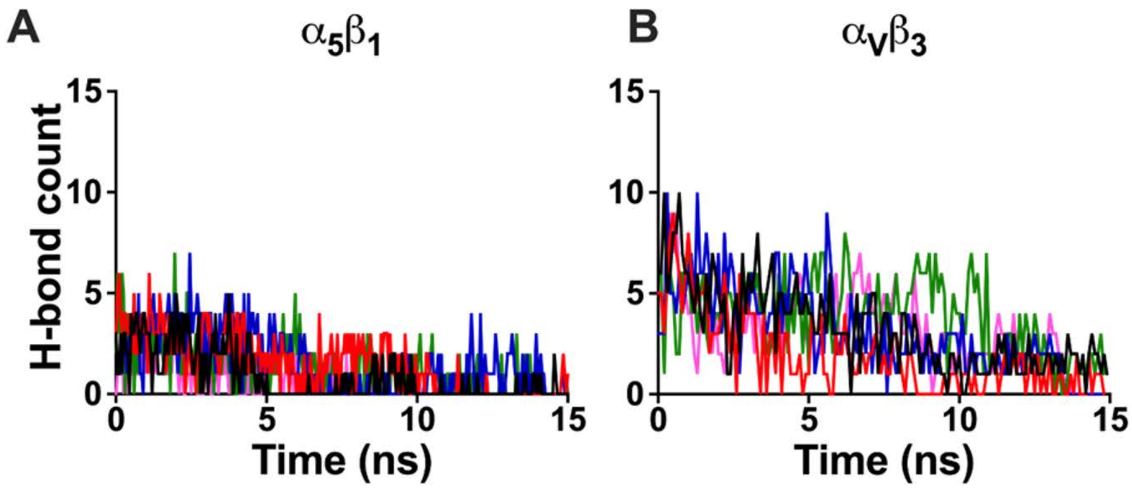

Supplementary figure 2. H-bond count of integrins $\alpha_5\beta_1$ (A) and $\alpha_V\beta_3$ (B) during unbending, collected from 5 independent runs of SMD simulations as indicated by different colors.

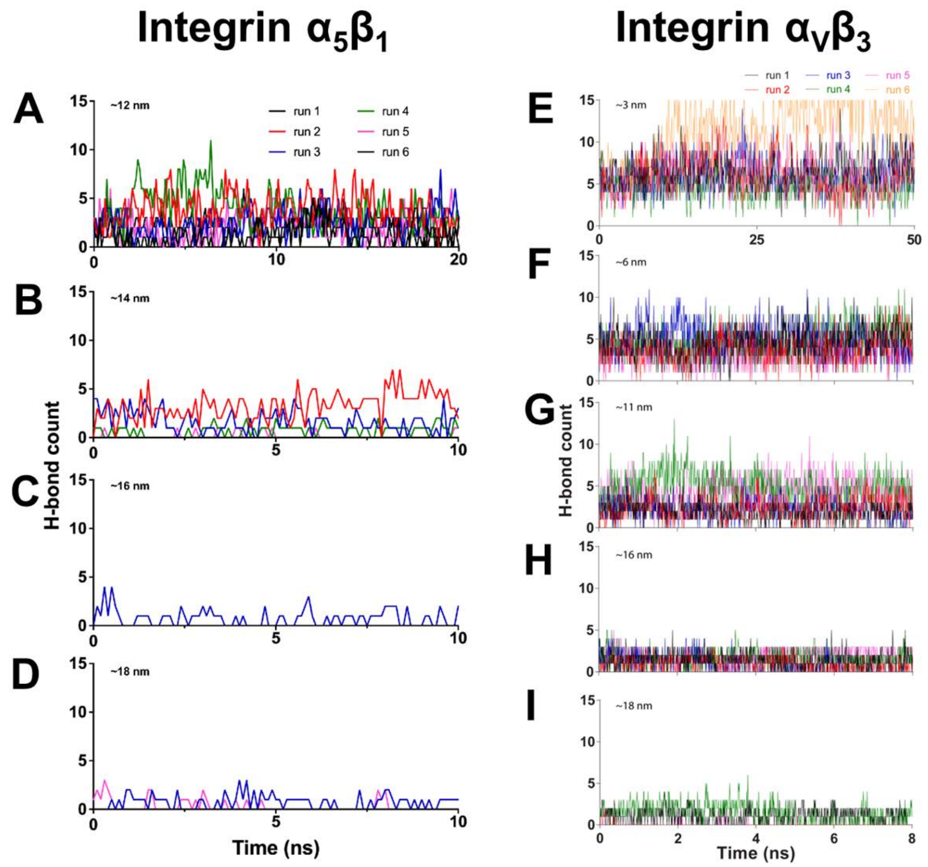

Supplementary figure 3. Using MD simulation to track the number of H-bonds between integrins $\alpha_5\beta_1$ and $\alpha_V\beta_3$ headpiece and tailpiece over time. For $\alpha_5\beta_1$, the integrin head-to-tail distance was free at ~12 nm (A) or restrained at ~14 (B), ~16 (C), and ~18 (D) nm, respectively. For $\alpha_V\beta_3$, the integrin head-to-tail distance was free at ~3 nm (E) or restrained at ~6 (F), ~11 (G), ~16 (H), and ~18 (I) nm, respectively. Each panel exhibits the traces of 6 repeated simulations, marked by different colors.

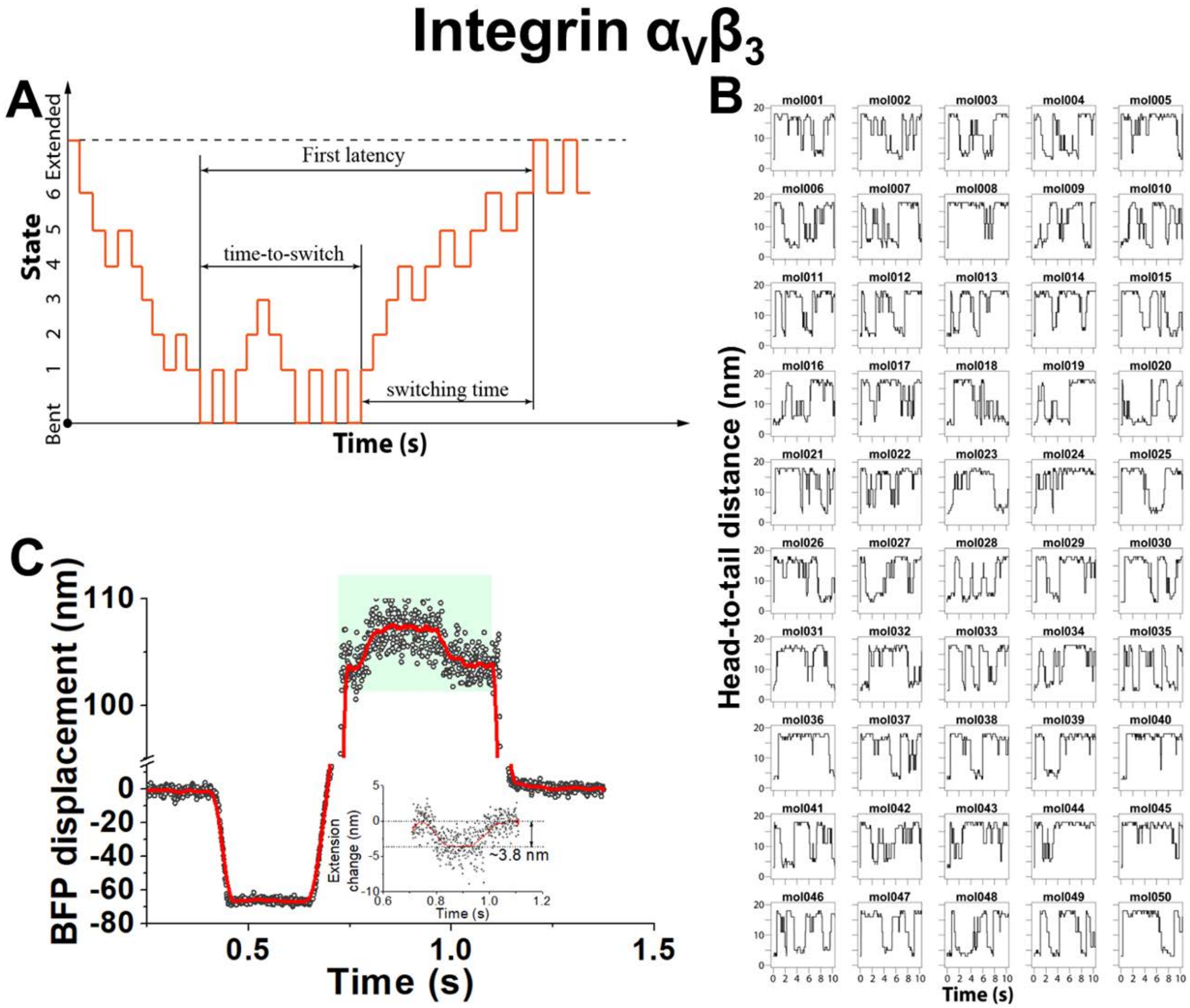

Supplementary figure 4. Simulation demonstration and experimental evidence of the multi-state model of integrin $\alpha_V\beta_3$ conformational changes. A. A representative 'mock run' of integrin $\alpha_V\beta_3$ conformational dynamics using Monte Carlo (MC) simulation, depicting an integrin fulfilling a bending event followed by an unbending event. Definition of time-to-switch and switching time of the unbending process were annotated on the graph. B. Integrin head-to-tail distance vs. time in 50 runs of MC simulation. Each panel represents one run. The head-to-tail distance was obtained based on the integrin's conformational state. C. A representative BFP force *vs*. time signal showing a partial and unfinished unbending event interrupted by an ensuing bending back.

Supplementary Tables

Supplementary table 1. Statistics of BFP-observed integrin $\alpha_V\beta_3$ lifetime events and unbending and bending events while binding to FN- and LIBS-2-coated probe beads.

Probe	Condition	#Lifetime events	Bent fraction	Extended fraction	Unbending		Bending	
					#Events	Probability	#Events	Probability
FN	Mn^{2+}	1424	0.37	0.63	185	35.11%	43	3.47%
	Ca^{2+}/Mg^{2+}	1847	0.75	0.25	164	11.84%	70	15.16%
	Mn^{2+}/NEM	106			8		0	
	Ca^{2+}/Mg^{2+}/ WOW-1	310	0.89	0.11	21	6.77%	3	8.72%
	Ca^{2+}/Mg^{2+}/ LIBS-2	183	0.20	0.80	5	13.46%	0	0%
LIBS-2	Ca^{2+}/Mg^{2+}	186			0		0	

Supplementary table 2. Fitted $f_{1/2}$ and Δx in the bending and unbending of different integrins and in different conditions using Eqs. 1 and 3. Fitting results of cell surface integrin $\alpha_V\beta_3$ were based on data from a previous publication[1].

	$\alpha_5\beta_1$		$\alpha_V\beta_3$, Ca^{2+}/Mg^{2+}		$\alpha_V\beta_3$, Mn^{2+}		$\alpha_V\beta_3$, cell, Ca^{2+}/Mg^{2+}	
	Eq. 1	Eq. 3	Eq. 1	Eq. 3	Eq. 1	Eq. 3	Eq. 1	Eq. 3
$f_{1/2}$ (pN)	7.4±0.6	6.04±0.01	3.5±0.6	5.9±0.6	-2.1±0.3	2.6±0.2	3.9±0.4	4.7±0.1
Δx (nm)	4.0±0.3	4.4±0.1	0.59±0.05	0.68±0.07	0.40±0.10	0.63±0.07	0.36±0.13	0.32±0.02

Supplementary table 3. Fitted kinetics and energy landscape parameters of integrin $\alpha_V\beta_3$ bending and unbending.

	k_+^{Bent} (s^{-1})	Δx_+^{Bent} (nm)	k_+ (s^{-1})	Δx_+ (nm)	k_-^{Extended} (s^{-1})	$\Delta x_-^{\text{Extended}}$ (nm)	k_- (s^{-1})	Δx_- (nm)
Ca^{2+}/ Mg^{2+}	1.93E+00	1.31E-01	4.84E+01	9.27E-02	2.13E+01	~0	3.83E+01	~0
Mn^{2+}	2.56E+00	3.57E-02	2.38E+01	1.01E-01	6.95E+00	~0	2.16E+01	~0

Supplementary Videos

Supplementary Video 1. Monte Carlo simulated transitions between the bent and extended conformations of integrin $\alpha_5\beta_1$ in the absence of externally applied force. Playback speed is 6.67-fold slower than real time.

Supplementary Video 2. Monte Carlo simulated transitions between the bent and extended conformations of integrin $\alpha_5\beta_1$ under 7.4 pN of pulling force. Playback speed is 6.67-fold slower than real time.

Supplementary Video 3. Monte Carlo simulated transitions between the bent and extended conformations of integrin $\alpha_5\beta_1$ under 15 pN of pulling force. Playback speed is 6.67-fold slower than real time.

Supplementary Video 4. Monte Carlo simulated transitions between the bent, intermediate, and extended conformations of integrin $\alpha_V\beta_3$ in the absence of externally applied force. Playback speed is 6.67-fold slower than real time.

Supplementary Video 5. Monte Carlo simulated transitions between the bent, intermediate, and extended conformations of integrin $\alpha_V\beta_3$ under 6 pN of pulling force. Playback speed is 6.67-fold slower than real time.

Supplementary Video 6. Monte Carlo simulated transitions between the bent, intermediate, and extended conformations of integrin $\alpha_V\beta_3$ under 12 pN of pulling force. Playback speed is 6.67-fold slower than real time.

Supplementary Methods

Solution of the multiple state transition kinetics

Since the transitions of integrins between different states (bent, intermediate and extended) are manipulated by stochastic thermodynamic energy, an integrin in any given intermediate state could transition towards both bending and unbending. We argue that the long switching time $t_{sw\pm}$ is essentially the add-up of the time-to-switch for all the jumps among intermediate states. By treating the stochastic conformational change as a Markov process in a finite state space, including bent, intermediate, and extended states. The dynamics of each state can be described as: $\frac{d\mathbf{S}}{dt} = \mathbf{TS}$, where **S** is the state occupancy vector. **T** is an N+2-by-N+2 matrix of transition rates, where N is the number of the intermediate states.

In the occupancy vector, S_{Bent}, S_i (i=1,2…N), and $S_{Extended}$ represent the bent, the ith intermediate, and the extended conformations. The transition rate matrix is defined by the transition rates:

$$T = \begin{pmatrix} -k_+^{\mathrm{Bent}} & k_-^1 & 0 & 0 & \cdots & 0 \\ k_+^{\mathrm{Bent}} & -\left(k_-^1 + k_+^1\right) & k_-^2 & 0 & \cdots & 0 \\ 0 & k_+^1 & -\left(k_-^2 + k_+^2\right) & k_-^3 & \cdots & 0 \\ & & \ddots & & & \\ \cdots & \cdots & \cdots & k_+^{\mathrm{N}-1} & -\left(k_-^{\mathrm{N}} + k_+^{\mathrm{N}}\right) & k_-^{\mathrm{Extended}} \\ 0 & \cdots & \cdots & 0 & k_+^{\mathrm{N}} & -k_-^{\mathrm{Extended}} \end{pmatrix} \qquad (6)$$

k_+^{Bent}, k_-^{Extended} are the rates of escaping from the bent or extended states to the nearby intermediate states. k_+^i, k_-^i (i=1,2…N) are respectively the transition rate of the ith intermediate state (indicated by the superscript) along the transition pathway. Marks "+" and "–" respectively represent the transition direction toward unbending and bending. All the other entries in **T** are equal to zero.

The mathematical definition of $t_{0\pm}$ and $t_{sw\pm}$ could then be given as follows. The sums of the time-to-switch $t_{0\pm}$ and switching time $t_{sw\pm}$ ($t_{0\pm}$+$t_{sw\pm}$) are the first latency to complete a full transition between two stable end-states (from bent to extended or *vice versa*; Supp. Fig. 4A). Notably, before the fulfillment of a complete transition, the molecule is allowed

to undergo multiple incomplete transitions ending by returning to the initial end-state. The $t_{0\pm}$ covers all the time consumed by these back-and-forth incomplete transitions, and finally when a complete transition starts, covers the latency for the end-state integrin to enter the first intermediate state (Supp. Fig. 4A). On the other hand, $t_{sw\pm}$ covers the rest of the time consumed by the molecule to complete the full transition, in which the molecule passes through all the intermediate states (mostly likely back-and-forth) and eventually arrives in the other end-state (Supp. Fig. 4A). By definition, a full transition requires the molecule to transit from one end-state to the other, during which the molecule cannot return to the initial end-state. Therefore, the first latency to complete transitioning from the intermediate state adjacent to end-state A to end-state B without returning to end-state A in the middle of the process is equal to the switching time $t_{sw\pm}$, which provides a simpler way to calculate $t_{sw\pm}$. In this case, the transition matrix is the submatrix of the original one, either deleting the first column and row or deleting the last column and row from the original matrix. The first latency to complete a full transition has been solved previously[2], where the average of the sum (<$t_{0\pm}$+$t_{sw\pm}$>) and the switching time (<$t_{sw\pm}$>) can be written as:

$$\begin{cases} \left\langle t_{0+} + t_{sw+} \right\rangle = \sum_{i=1}^{\mathrm{N}} \dfrac{1+\sum_{j=i}^{\mathrm{N}} \prod_{k=i}^{j} r_k}{k_+^{i\text{-}1}} + \dfrac{1}{k_+^{\mathrm{N}}} \\ \left\langle t_{0-} + t_{sw-} \right\rangle = \sum_{i=1}^{\mathrm{N}} \dfrac{1+\sum_{j=1}^{i} \prod_{k=j}^{i} \dfrac{1}{r_k}}{k_+^{i+1}} + \dfrac{1}{k_-^{1}} \\ \left\langle t_{sw+} \right\rangle = \sum_{i=2}^{\mathrm{N}} \dfrac{1+\sum_{j=i}^{\mathrm{N}} \prod_{k=i}^{j} r_k}{k_+^{i\text{-}1}} + \dfrac{1}{k_+^{\mathrm{N}}} \\ \left\langle t_{sw-} \right\rangle = \sum_{i=1}^{\mathrm{N\text{-}1}} \dfrac{1+\sum_{j=1}^{i} \prod_{k=j}^{i} \dfrac{1}{r_k}}{k_+^{i+1}} + \dfrac{1}{k_-^{1}} \end{cases} \quad (7)$$

where $r_i = \dfrac{k_-^i}{k_+^i}$, $k_+^0 = k_+^{\mathrm{Bent}}$, and $k_-^{N+1} = k_-^{\mathrm{Extended}}$

By subtracting the switching time from the first latency to complete a full transition, one can obtain the time-to-switch. Thus, the time-to-switch and switching time can be summarized as below:

$$\begin{cases} \langle t_{0+} \rangle = \frac{1+\sum_{j=1}^{N} \prod_{k=1}^{j} r_k}{k_{+}^{\text{Bent}}} \\ \langle t_{0-} \rangle = \frac{1+\sum_{j=1}^{N} \prod_{k=j}^{N} \frac{1}{r_k}}{k_{-}^{\text{Extended}}} \\ \langle t_{sw+} \rangle = \sum_{i=2}^{N} \frac{1+\sum_{j=i}^{N} \prod_{k=i}^{j} r_k}{k_{+}^{i\text{-}1}} + \frac{1}{k_{+}^{N}} \\ \langle t_{sw-} \rangle = \sum_{i=1}^{\text{N-1}} \frac{1+\sum_{j=1}^{i} \prod_{k=j}^{i} \frac{1}{r_k}}{k_{+}^{i+1}} + \frac{1}{k_{-}^{1}} \end{cases} \tag{8}$$

Each energy barrier on the transition pathway corresponded to the breakage/formation of a H-bond. To simplify our model, we assumed all the energy barriers among the intermediate states to be identical. Therefore, the transition rates k_{+}^{i}, k_{-}^{i} (i=1, 2…N) were identical between every two neighboring states, and were simply labeled as k_{-} and k_{+}, respectively. In turn, all the ratios of transition rates, $r_i = \frac{k_{-}^{i}}{k_{+}^{i}}$, were also identical and simply labeled as r. With these simplifications, we rewrote the model as Eq. 4.

Supplementary references:

1 Chen, Y., Lee, H., Tong, H., Schwartz, M. & Zhu, C. Force regulated conformational change of integrin alphaVbeta3. *Matrix biology* **60-61**, 70-85, doi:10.1016/j.matbio.2016.07.002 (2017).

2 Zhou, Y., Pearson, J. E. & Auerbach, A. Phi-value analysis of a linear, sequential reaction mechanism: theory and application to ion channel gating. *Biophysical journal* **89**, 3680-3685, doi:10.1529/biophysj.105.067215 (2005).
